# Informal and formal long-term care utilization and unmet needs in Europe: examining socioeconomic disparities and the role of social policies for older adults

**DOI:** 10.1007/s10754-024-09378-z

**Published:** 2024-05-21

**Authors:** Viktoria Szenkurök, Daniela Weber, Marcel Bilger

**Affiliations:** 1 Health Economics and Policy, Vienna University of Economics and Business, Vienna, Austria; 2 Population and Just Societies Program, International Institute for Applied Systems Analysis (IIASA), Wittgenstein Centre for Demography and Global Human Capital (IIASA, OeAW, Univ. Vienna), Laxenburg, Austria

**Keywords:** Long-term care, SHARE, Two-part multilevel model, Socioeconomic status, Care policy, Pension generosity, I14, I18, J18

## Abstract

The rising number of older adults with limitations in their daily activities has major implications for the demands placed on long-term care (LTC) systems across Europe. Recognizing that demand can be both constrained and encouraged by individual and country-specific factors, this study explains the uptake of home-based long-term care in 18 European countries with LTC policies and pension generosity along with individual factors such as socioeconomic status. Using data from the Survey of Health, Ageing and Retirement in Europe conducted in 2019, we apply a two-part multilevel model to assess if disparities in use of LTC are driven by disparities in needs or disparities in use of care when in need. While individual characteristics largely affect the use of care through its association with disparities in need, country-level characteristics are important for the use of care when in need. In particular, the better health of wealthier and more educated individuals makes them less likely to use any type of home-based personal care. At the country level, results show that the absence of a means-tested benefit scheme and the availability of cash-for-care benefits (as opposed to in-kind) are strongly associated with the use of formal care, whether it is mixed (with informal care) or exclusive. LTC policies are, however, shown to be insufficient to significantly reduce unmet needs for personal care. Conversely, generous pensions are significantly associated with lower unmet needs, underscoring the importance of considering the likely adverse effects of future pension reforms.

**Supplementary Information:**

The online version contains supplementary material available at 10.1007/s10754-024-09378-z.

## Introduction

According to a 2021 report by the European Commission and Directorate-General for Employment, Social Affairs and Inclusion (2021), it was projected that the absolute number of individuals reporting care needs would surge from 30.8 million in 2019 to 38.1 million by 2050. This trend aligns with the *expansion of morbidity* hypothesis first developed by Gruenberg (1977), advocating the pessimistic view that the observed increase in life expectancy is predominantly due to a decline in the mortality rate of diseases rather than a reduction in their prevalence. Correspondingly, the growing population facing limitations in activities of daily living (ADL) is straining care systems across the world (Scherbov & Weber, 2017). One possibility to address these issues is to promote home-based care, as this has not only been shown to be more cost-effective than residential care (Colombo et al., 2011; Da Roit & Le Bihan, 2010; European Commission et al., 2018; Kok et al., 2015), but also to be preferred over residential care by older adults (Kristinsdottir et al., 2021; Tarricone & Tsouros, 2008).

As people with limitations in their daily activities face individual (e.g. limited social or economic resources) as well as institutional (e.g. existing welfare state arrangements) constraints, access to and utilization of home-based long-term care (LTC) is likely to vary between individuals and countries likewise (European Commission and Directorate-General for Employment, Social Affairs and Inclusion, 2021).

A vast majority of studies explaining disparities in the use of home-based care has focused on the role of individual characteristics, especially socioeconomic status, which was most commonly defined by income, wealth or, less commonly, education (Albertini & Pavolini, 2017; Broese van Groenou et al., 2006; Lera et al., 2021). Yet, no final consensus has been made on how socioeconomic status affects the use of LTC when in need. Several studies found that while formal care use is more prevalent among the rich, informal care is more present among the poor (Albertini & Pavolini, 2017; García-Gómez et al., 2015; Ilinca et al., 2017; Lera et al., 2021; Rodrigues et al., 2018; Tenand et al., 2020a, 2020b). Once combinations of formal and informal care were included, results varied. Broese van Groenou et al. (2006) showed that the educational gradient in the utilization of both formal and informal care exists, and that differences in age, health and marital status largely account for the former but not the latter. Conversely, Suanet et al. (2012) did not find a significant effect of education on mixed home-based care use. Floridi et al. (2020) reported that income has a stronger association with formal care, while wealth has a stronger association with informal and mixed care.

Disparities in the uptake of home care may also depend on country-specific institutions (Ranci & Pavolini, 2013). Countries with a traditional family-based approach to care have sought to increase choice by introducing care allowances and removing persisting access barriers such as means testing. Others, taking a de-familiaristic approach, have focused on strengthening the responsibility of the state by further expanding access to formal and institutionalized care services. However, countries that heavily rely on the exclusive use of either informal or formal care might face problems in the near future once the availability of informal (and formal) carers, and their willingness to provide care, declines (Agree & Glaser, 2009; European Commission and Directorate-General for Employment, Social Affairs and Inclusion, 2021; Hassink & Van den Berg, 2011). In addition, norms of reciprocity and solidarity are the drivers of informal care, particularly at the family level, but changes in the labor market, including the rise in female labor force participation and the modification of pension systems, may further exacerbate issues in meeting care needs appropriately (Bengtson & Roberts, 1991). Selected studies suggested that aging populations in countries with more comprehensive welfare state arrangements often receive a combination of informal and formal care (Motel-Klingebiel et al., 2005; Suanet et al., 2012). Further, socioeconomic inequalities in the use of mixed care are lower in more de-familized care systems using the number of LTC beds as a proxy for de-familization (Floridi et al., 2020). However, the empirical literature on the study of disparities in the use of LTC services suffers from two deficiencies. First, despite some studies highlighting the importance of contextualizing findings in light of their country-specific characteristics including LTC policies (Albuquerque, 2022), limited research has been conducted on relevant social policies for older adults (e.g., LTC and pension policies) and their respective role in explaining informal and formal care use. Second, despite selected single-country studies using horizontal inequity indices to analyze need-standardized LTC use (e.g. Tenand et al., 2020a), no particular attention has been paid to potential effects of disparities in the need for care. Instead, the literature examining disparities in utilization emphasizes the study of those with preexisting needs. While this is important, it ignores that (socioeconomic) disparities in need may contribute to and potentially exacerbate disparities in use. Taking these into account is crucial, especially if we are to derive appropriate policy measures.

This study aims at filling these gaps in the literature by analyzing cross-sectional data from the eighth wave of the Survey of Health, Ageing and Retirement in Europe (SHARE) and combining it with country-level data on the design and nature of national LTC policies and generosity of the pension scheme. Using a two-part multilevel approach, we explain disparities in the use of informal and formal home-based care using individual socioeconomic status and country-level institutional arrangements in 18 European countries. While, at the individual level, we focus on the study of social, material and human resources, at the country level, we differentiate between LTC policies on the one hand (captured by the availability of cash-for-care benefits, restrictions on acquiring these or other LTC benefits through the application of means testing and the state’s responsibility for care) and pension generosity on the other.

Our study hence differs in two important ways from previous analyzes that focus on disparities in the use of home-based LTC services. First, unlike previous research, we deliberately do not restrict our sample to individuals who are more likely to have personal care needs. Instead, we endeavor to explain the use of home-based care for older adults, considering that both individual-level and country-level characteristics may play an influential role in having needs. Second, this work expands previous knowledge on the utilization and distribution of older adult’s long-term care as it encompasses and examines a comprehensive range of social policies for older adults for a uniquely large set of 18 European countries. To be able to include so many countries in the analysis, we focused on the most recent SHARE wave available. Such cross-section analysis is justified by the circumstance that LTC policies are geographically very heterogeneous with little change in recent years. With this approach we are not only able to provide new insights into potential drivers of disparities in use of care and unmet needs, but also provide a solid foundation for policy action to mitigate these.

The remainder of this article is organized as follows. First, the data and method will be presented in Section “Methods”. Followed by a discussion in Section “Discussion”, in Section “Results” we report our main results. Concluding remarks are given in Section “Conclusion”.

## Methods

### Data

We use cross-sectional data from the eighth wave of the Survey of Health, Ageing and Retirement in Europe (SHARE) conducted in 2019/2020 (Börsch-Supan, 2022; Börsch-Supan et al., 2013). SHARE is a multidisciplinary survey nationally representative of non-institutionalized adults aged at least 50 years that includes information on respondents’ demographic, socioeconomic, health characteristics, as well as their use of home care from both informal and formal sources. Further, we use country-level information from OECD Statistics and the Mutual Information System on Social Protection, which includes information on social protection systems and their organization (MISSOC, 2022; OECD, 2021a). Our study sample includes 35,547 survey participants from 18 European countries with available country-level data (see sample statistics by country in the supplementary material).

#### Dependent variables

*Informal and formal* home-based *care utilization* in the past 12 months was self-reported (Carrieri et al., 2017; Floridi et al., 2020; Ilinca et al., 2017). Here, personal care may include help with tasks such as dressing, walking, and eating but excludes practical household help such as home repairs or help with paperwork. We distinguish four categories: (i) *no care* utilization (i.e. reference category), (ii) exclusively *informal care* by family members or non-family members inside or outside the household, (iii) exclusively *formal care* in the form of professional or paid personal care services, and (iv) *mixed care* a mixture of informal and formal home care.

We capture *personal care need*s by two commonly-used measures of self-reported functioning limitations, which we consider as upper and lower boundaries of care need. Activities of daily living (ADL) show limitations in vital skills by recording differences with *“bathing”, “dressing”, “grooming”, “eating”, “transferring”* and *“toileting”.* Instrumental activities of daily living (IADL) summarize important competencies required for living independently in a community. Any difficulties in doing the everyday activities *“doing work around the house or garden”, “leaving the house independently/accessing transportation”, “shopping for groceries”, “doing personal laundry”, “managing money”, “preparing a hot meal”, “taking medication”* and *“making telephone calls”* are recorded (Portela et al., 2020). For both variables, we distinguish two categories (i) *no limitations* (i.e., reference category) and (ii) at least one limitation with (I)ADL.

#### Individual-level characteristics

Following the theoretical frameworks by Grossman (1972) and Andersen and Newman (1973), we suggest that disparities in the probability to use informal and formal care are related to socioeconomic inequalities. The disparities in care use include care need as well as one’s opportunity to access care when in need (Broese van Groenou et al., 2006; van der Meer, 1998). Here, socioeconomic status is captured by the availability of social resources (i.e., the presence of a spouse or children), financial and material resources (i.e., household net wealth), and human resources (i.e., educational attainment classified by ISCED). We rely on household net wealth as it includes both financial assets and real assets (e.g., home ownership), and is thus better able to reflect the social status of older adults. Further, wealth captures the cumulative impact of lifelong advantages and disadvantages in terms of material resources (Kaplan et al., 1987; Robert & House, 1996). As sensitivity test, results using household net income instead of wealth are enclosed in the supplementary material (see Table S.14). To address the common issue of a large number of missing values of monetary variables due to non-response, SHARE data provide imputations for both household net income and household net worth (i.e., wealth) using the fully conditional specification (FCS) method (Börsch-Supan, 2022; van Buuren et al., 1999). In addition, we include predisposing demographic factors such as age and gender.

#### Country-level characteristics

Drawing on Andersen and Newman’s behavioral model of health care utilization and van Groenou and De Boer’s model of informal care, we focus on social policies for older adults across two main domains: (i) the LTC system and (ii) the pension scheme (Andersen & Newman, 1973; Broese van Groenou & De Boer, 2016).

The first domain captures LTC-related characteristics, including the design (i.e., the availability of cash-for care services, the application of means testing) and the responsibility of the state to meet LTC needs. For the former, we have created binary indicators that map the availability of cash-for-care benefits (with in-kind benefits as the reference category) and the application of a means test in meeting the requirements for receiving care-related benefits. Data for these two indicators are sourced from Ariaans et al. (2021) and have been updated with information from MISSOC (MISSOC, 2022). In addition we use the availability of beds in long-term inpatient care beds per 1000 inhabitants aged 65 and older to capture the state’s responsibility to provide for LTC (Floridi et al., 2020; OECD, 2021a).

The second domain captures the generosity of the pension scheme measured by the net pension entitlement divided by net pre-retirement earnings, taking into account personal income taxes and social security contributions paid by workers and pensioners (i.e., net replacement rate) (OECD, 2022).

We further control for macroeconomic factors including the GDP per capita, the consumer price index for personal care and the female labor market participation rate (OECD, 2021b, 2021c).

### Estimation strategy

In this study, we analyze disparities in the consumption of long-term care while taking also the need for care into account. We do so by adopting a two-part multilevel design given the hierarchical nature of the data. The first part describes the probability of being in need of personal care whereas the second part describes the probability of using formal, informal or mixed care conditional on reporting personal care needs. This decomposition of our model into two parts builds on the assumption that if need is observed, then some form of care will be required (Belotti et al., 2015; Oshchepkov & Shirokanova, 2020).

#### Need for personal care (Part I)

In this first part, we assume that if functional limitations are reported, some form of (home-based) LTC is needed. By using a binary multilevel logit regression with random intercepts, which allow for the average level of each type of care to vary randomly across individuals within countries, we first examined the probability of being in need of personal care using our set of individual and country-level characteristics as explanatory variables. That is:

$$Pr\left({y}_{ij}^{*}>0 |{x}_{ij},{u}_{0j}\right)=\text{Pr}\left({\gamma }_{00}+ {\gamma }_{10}{IC}_{ij}+ {\gamma }_{10}{CC}_{j}+{u}_{0j} +{\varepsilon }_{ij}>0 \right|{u}_{0j}),$$

where $${x}_{ij}$$ is a vector of explanatory variables that contain individual-level ($${IC}_{ij})$$ and country-level characteristics $${(CC}_{j})$$ of individual *i* in country* j*. We assume that $${u}_{0j}$$ is distributed as $$N\left(0,{\sigma }_{{u}_{0}}^{2}\right)$$ and the cumulative distribution of $${\varepsilon }_{ij}$$ is assumed to be logistic (Belotti et al., 2015).

#### Care use conditional on need (Part II)

The second part of the model explains care use of the subpopulation reporting care needs. Conditional on reporting personal care needs in Part I *(*i.e*.,*${y}_{ij}^{*}$ > 0), a multinomial random intercept multilevel logit regression was applied to determine the probability of actually using care. We formulated the relationship as the following:

$$E\left( {y_{ij} | y_{ij}^{*} > 0,x_{ij} ,u_{0j} } \right) = \Pr {(}\gamma_{00} + \gamma_{10} IC_{ij} + \gamma_{10} CC_{j} + u_{0j} + \varepsilon_{ij} {|}y_{ij}^{*} > 0,u_{0j}),$$

where ${y}_{ij}$ denotes one of four possible options of care utilization (i.e., no care, informal care, formal care or mixed care) of individual *i* in country *j.*

#### Unconditional care use

In the full model, which combines both parts, we explain care use for the whole sample population. The decomposition of disparities into two parts aimed at identifying cumulative disparities in care use ($\hat{y}_{ij}$) unconditional on care need. In mathematical terms, the overall mean can be written as the product of predictions from the first and second part of the model (i.e., *Part I* and *Part II*), which is:

$$E\left( {\hat{y}_{ij} |x_{ij} } \right) = \Pr \left( {y_{ij}^{*} > 0|x_{ij} } \right) \times E\left( {y_{ij} | y_{ij}^{*} > 0,x_{ij} } \right)$$

The standard errors of the average marginal effects reported in Section “Results” are calculated using bootstrap (with 100 sample replications). The bootstrap method generates bootstrap samples to fit our two-part model, and get variances and inverted bootstrap confidence intervals of predictive margins (Duan et al., 2022).

### Robustness and sensitivity analysis

We used different severity thresholds of both IADL and ADL (e.g. at least 2 limitations) to test the sensitivity of our definition of ‘in need of care’. As Roquebert et al (2021) suggests that results may vary depending on whether we rely on objective or subjective indicators, with formal care utilisation potentially being more sensitive to subjective measures than informal care, we also used the comparatively more subjective Global Activity Limitation Indicator (GALI) to define ‘in need of care’ (Tinios & Valvis, 2023). GALI equally takes into account the severity of need, distinguishing between mild and severe limitations, yet the validity to accurately categorize the degree of severity of an individual’s disability is limited (see supplementary material) (Van Oyen et al., 2018). (I)ADL serve not only as common eligibility criterion for LTC related benefits, in-kind and in cash, but they are also preferred over more subjective measures of need including the GALI (European Commission and Directorate-General for Employment, Social Affairs and Inclusion, 2021; Tarazona et al., 2021).

Since there may also be gender-specific differences in the influence of individual and country-specific characteristics on the need for care as well as the use of care, we run the described models separately for women and men. Results of this gender-specific analysis are reported in the supplementary material and show marginal effects, allowing for a proper comparison between both subsamples.

## Results

### Descriptive results

**Fig. 1:**
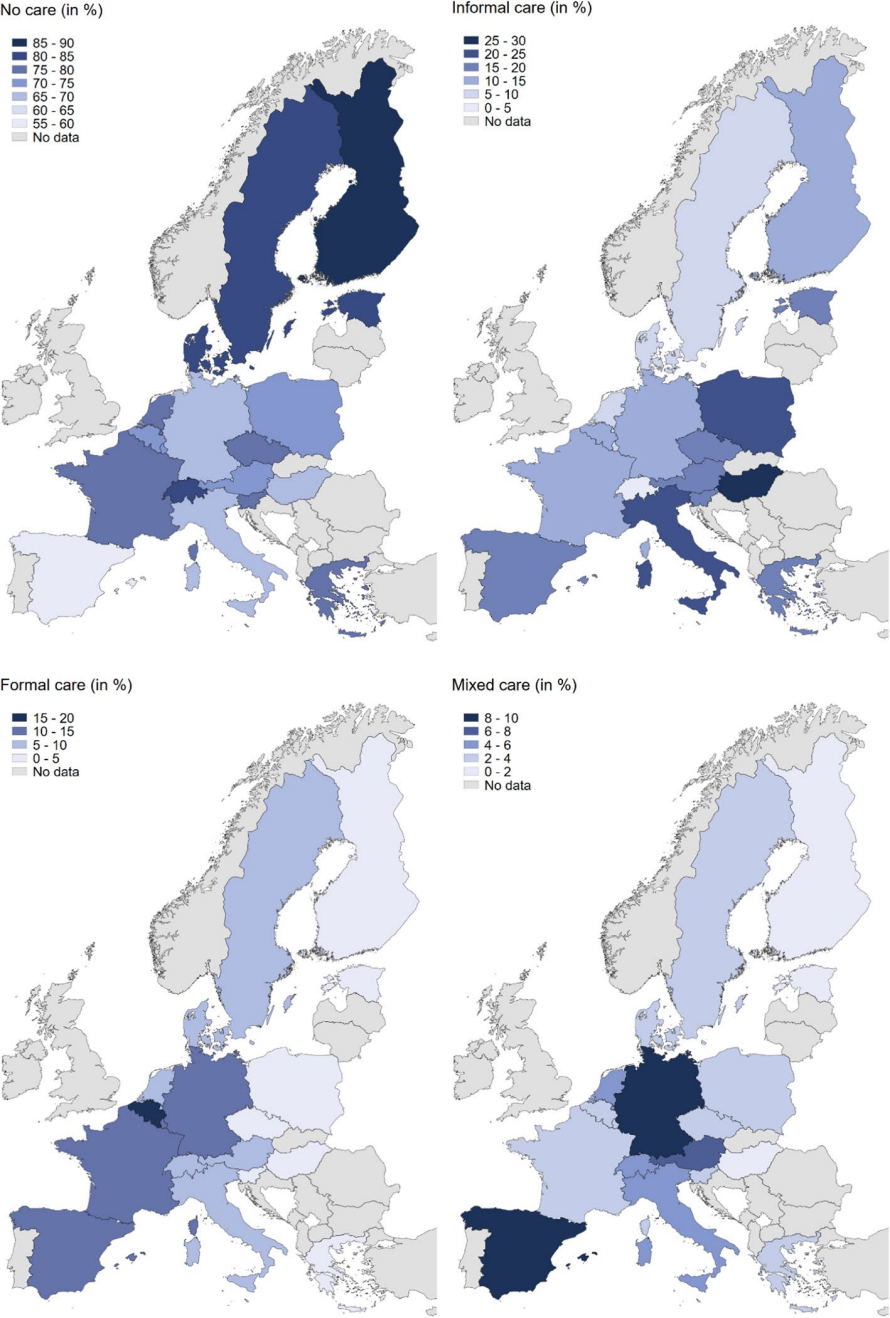
Care usage (no care, informal, formal and mixed care) among older adults with care needs in 2019/2020 in Europe

Figure 1 shows that informal care is the predominant type of care compared to formal or mixed care, yet the extent to which a country resorts to informal care varies considerably across Europe. While the prevalence of informal care is relatively high in Southern and Eastern European countries, Western and Northern European countries increasingly resort to formal care when limitations with IADL are reported. Austria, Germany and Spain belong to the countries with the highest prevalence of mixed care, with a share between 6 and 10%. No less important is the clear North–South divide in the proportion of people who do not receive personal care although limitations exist. In Denmark, Finland and Sweden the respective share varies between 80 and 90%, while in Italy and Spain it remains between only 55 and 65%.

Descriptive statistics in total and by care utilization (see Table 1) suggest that, compared with formal or mixed care users, individuals receiving only informal care (3.2% of the whole sample) report more social resources (i.e., spouse, partner or children). Individuals receiving formal (1.9%) or mixed care (0.8%) are on average older and disproportionately female, but they also report more disabilities (i.e., IADL and ADL). Of all care recipients, the proportion of older adults with the highest level of education is largest among those receiving mixed care, and lowest among those receiving only informal care. Moreover, older adults using formal or mixed care are more likely to live in countries where state responsibility is high (i.e., LTC beds), cash-for-care benefits are available and LTC related benefits are means-tested. Similarly, the generosity of pensions (i.e., net replacement rate) is highest among those receiving formal care.

**Table 1:** Descriptive statistics—Average care need prevalence, individual- and country-level characteristics, by type of care use and in total

	(1)	(2)	(3)	(4)	(5)
	No care	Informal care	Formal care	Mixed care	Total
**Care need**					
*IADL (Instrumental Activities of Daily Living)*					
At least 1 (%)	12.4	85.9***	87.5***	98.0***	16.2
At least 2 (%)	5.8	69.6***	74.3***	92.5***	9.3
*ADL (Activities of Daily Living)*					
At least 1 (%)	7.3	83.9***	77.7***	95.7***	11.1
At least 2 (%)	2.9	48.7***	59.3***	77.8***	5.5
**Individual-level characteristics**					
Socioeconomic status					
*Material resources (%)*					
2nd wealth quintile	19.2	22.8***	19.2***	22.7**	19.4
3rd wealth quintile	18.8	17.2***	15.9***	22.2***	18.7
4th wealth quintile	20.7	13.4***	14.9***	15.9***	20.4
5th wealth quintile	21.2	13.5***	6.7***	8.8***	20.6
*Social resources (%)*					
Has a spouse/partner	67.5	74.7***	33.8***	71.6**	67.2
Has children	88.4	89.9**	80.6***	91.5	88.3
*Human resources (%)*					
Secondary education	61.9	55.7***	54.5***	49.6***	61.5
Tertiary education	22.4	10.9***	11.4***	14.5***	21.9
Predisposing factors					
Age (years)	66.0	74.8***	81.3***	79.2***	66.6
Female (%)	52.7	52.5***	67.8***	57.1**	53.0
**Country-level characteristics**					
LTC policy					
Means testing (%)	50.2	51.8	60.5	52.3	50.5
Cash-for-care benefits^a^ (%)	69.8	69.3	76.6***	81.0***	70.1
LTC beds (p. 1,000)	44.9	40.4***	51.4***	45.4	44.9
Pension generosity					
Replacement rate (%)	63.9	64.6	67.8***	66.9***	64.1
Macroeconomic factors					
Consumer price index (personal care)	102.8	103.1**	102.7	102.6	102.8
GDP p. capita (in PPS)	107.5	97.2***	114.4***	107.6	107.3
Female labor market part. (%)	70.7	69.1***	70.8	70.5	70.7
N	33,437	1139	671	300	35,547

Statistical differences (*p < 0.10, **p < 0.05, ***p < 0.01) are reported using “no care” as reference category. SHARE data include survey weights

^a^Reference category denotes in-kind benefits

### Two-part model results

Calculated average marginal effects from the two-part regression analysis show that both individual and country characteristics play a crucial role in explaining disparities in the probability of using (in)formal LTC services (see Table 2; columns 5–7). By decomposing our model into two parts*, Part I* and *Part II* (i.e., columns 1 and 2–4), we are able to gain a better understanding of the drivers of these outcomes. In a nutshell, while disparities in use are strongly driven by individual-level disparities in having care needs (i.e., *Part I*), the national design of LTC policies and the generosity of the pension scheme are important explanatory factors for the demand for (in)formal care when needed (i.e., *Part II)*. Using McFadden’s pseudo R-squared to indicate the explained variance in the outcome variable across both parts of the model reinforces this observation. Overall, individual and country-level variables account for 14.1% of the variance (McFadden pseudo R^2^ 0.141) in need (Part I), while it is 8.8% of the variance (McFadden pseudo R^2^ 0.088) in care utilization (Part II). Further, it shows that the inclusion of country-level variables alongside individual-level ones contributes to explaining an additional 2.4% of the variance in care use in Part II (McFadden pseudo R^2^ 0.024). This is notably higher than the mere 0.1% observed in Part I (McFadden pseudo R^2^ 0.005), emphasizing their distinct role in explaining need and use of care (see Table 2).

**Table 2:** Probability of care need (1 + IADL) and utilization by care type-individual- and country-level average marginal effects

	(Part I) Need for personal care	(Part II) Care use conditional on need			Unconditional care use		
	*(1)* *1* + *IADL*	*(2)* *informal care*	*(3)* *formal care*	*(4)* *mixed care*	*(5)* *informal care*	*(6)* *formal care*	*(7)* *mixed care*
**Individual-level characteristics**							
Socioeconomic status							
*Material resources (ref. 1st wealth quintile)*							
2nd wealth quintile	− 0.032*** (0.006)	− 0.027** (0.012)	− 0.015* (0.008)	− 0.012* (0.007)	− 0.120*** (0.034)	− 0.108*** (0.032)	− 0.137** (0.058)
3rd wealth quintile	− 0.070*** (0.006)	− 0.015 (0.013)	− 0.015 (0.009)	− 0.009 (0.007)	− 0.125*** (0.036)	− 0.121*** (0.046)	− 0.112* (0.062)
4th wealth quintile	− 0.077*** (0.007)	− 0.031** (0.013)	− 0.024** (0.009)	− 0.018** (0.007)	− 0.190*** (0.040)	− 0.186*** (0.047)	− 0.211*** (0.063)
5th wealth quintile	− 0.100*** (0.007)	− 0.027* (0.014)	− 0.047*** (0.227)	− 0.008 (0.009)	− 0.208*** (0.037)	− 0.357*** (0.082)	− 0.133** (0.060)
*Human resources (ref. primary education)*							
Secondary education	− 0.061*** (0.006)	0.007 (0.011)	− 0.010 (0.007)	− 0.008 (0.006)	− 0.051 (0.033)	− 0.086** (0.038)	− 0.087* (0.049)
Tertiary education	− 0.107*** (0.007)	0.003 (0.014)	− 0.014 (0.010)	− 0.011 (0.008)	− 0.121*** (0.044)	− 0.136** (0.055)	− 0.127* (0.072)
*Social resources*							
Has spouse/partner	− 0.007 (0.004)	0.125*** (0.009)	− 0.039*** (0.007)	0.040*** (0.005)	0.377*** (0.027)	− 0.125*** (0.034)	0.408*** (0.056)
Has children	− 0.039*** (0.008)	0.027* (0.014)	− 0.042*** (0.012)	− 0.000 (0.009)	0.022 (0.051)	− 0.195*** (0.049)	− 0.023 (0.075)
Predisposing factors							
Age	− 0.034*** (0.003)	− 0.000 (0.006)	− 0.003 (0.005)	− 0.006* (0.003)	− 0.044** (0.019)	− 0.039* (0.023)	− 0.064** (0.028)
Age^2^	0.00030*** (0.000)	0.00001 (0.000)	0.00005 (0.000)	0.00005** (0.000)	0. 00042*** (0.000)	.00045*** (0.000)	0.00055*** (0.000)
Gender (female)	0.052*** (0.004)	− 0.046*** (0.009)	− 0.003 (0.007)	− 0.012** (0.005)	− 0.075*** (0.027)	− 0.007 (0.033)	− 0.107*** (0.041)
**Country-level characteristics**							
LTC policy							
Means testing (ref. none)	− 0.020 (0.017)	0.005 (0.018)	− 0.037*** (0.013)	− 0.010 (0.008)	− 0.029 (0.033)	− 0.196*** (0.048)	− 0.104* (0.054)
Cash-for-care benefits (ref. in-kind benefits)	− 0.003 (0.016)	0.015 (0.017)	0.003 (0.011)	0.021*** (0.006)	0.054 (0.036)	0.031 (0.042)	0.212*** (0.055)
LTC beds (p. 1000)	0.002** (0.001)	− 0.000 (0.001)	0.003*** (0.001)	− 0.000 (0.000)	0.002 (0.002)	0.014*** (0.002)	0.001 (0.003)
Pension generosity							
net replacement rate (%)	0.000 (0.000)	0.001 (0.000)	0.001** (0.000)	0.000 (0.000)	0.003*** (0.001)	0.004*** (0.001)	0.003** (0.001)
Macroeconomic factors							
Consumer price index (personal care)	0.016 (0.014)	0.005** (0.002)	− 0.004*** (0.020)	− 0.001 (0.001)	0.012*** (0.003)	− 0.020*** (0.005)	− 0.007 (0.006)
GDP p. capita (in PPS)	− 0.125*** (0.039)	− 0.190*** (0.050)	0.012 (0.026)	0.008 (0.020)	− 0.669*** (0.127)	− 0.083 (0.113)	− 0.076 (0.119)
Female labor market part. (%)	− 0.002 (0.002)	− 0.002 (0.002)	− 0.003*** (0.016)	0.001 (0.001)	− 0.010*** (0.004)	− 0.017*** (0.004)	0.005 (0.006)
N	35,547	6959			35,547		

(Bootstrapped) standard errors are in parentheses

*p < 0.10, **p < 0.05, ***p < 0.01

#### Utilization of informal, formal and mixed care

Our results show that wealth is associated with significantly less care use, with exclusive forms being particularly affected (i.e., columns 5–7 in Table 2). This association between the unconditional probability to use informal or formal care and wealth is strongly driven by a wealth gradient in need (i.e., column 1 in Table 2). Moreover, when needs are reported, wealthier individuals are less likely to use personal care (i.e., column 2–4 in Table 2). Statistics on the severity of limitations in each wealth quintile reveal that wealthy individuals report fewer limitations than their counterparts and may therefore be more able to substitute personal care with other sources such as practical household help (see Table S.6 in supplementary material). Similarly, observable educational gradients in care use are strongly determined by a gradient in the need for care. However, in contrast to wealth, results suggest that education plays a lesser role in explaining care utilization when in need. In contrast to human resources, social resources play a key role when there is need. Results from our analysis reveal that having a partner is positively associated with informal care use (both exclusive and mixed) (Table 2). We also find that children, albeit less strongly than wealth or education, contribute to reducing the likelihood of needing care.

A separate analysis reveals that this result also has a strong gender dimension (see Table S.8–S.11 in supplementary material). Interestingly, we find that the effect of a spouse or partner on informal care (when in need) is significantly greater for men than for women. Overall, gender differences in use are clearly visible. Results show that women are more likely to be in need, but less likely to use (informal) care when in need. The observable (non-linear) effect of age is confirmatory.

While socioeconomic characteristics influence disparities in the use of care services mainly through their impact on need, institutional factors are shown to be particularly influential in meeting existing needs by influencing the choice between forms of care when needed. Older adults living in countries applying means testing for care-related benefits report significantly lower uptake of formal care services at home (both exclusive and mixed). However, the availability of cash-for-care benefits (as opposed to in-kind benefits only) is associated with significantly higher utilization of mixed care. Both effects on the unconditional probability to use care are driven by disparities in use when having needs (columns 2–4 in Table 2). Overall, state support (measured by the number of LTC beds available) is positively associated with formal care use. More state support, however, is associated with a greater likelihood to have needs. When having needs, state support contributes positively in meeting those needs formally. Results ﻿further indicate that generous pensions are associated with higher uptake, regardless of the type of personal care. The impact on the unconditional probability is, however, greatest for formal care﻿.

The marginal effects of macroeconomic variables such as consumer price and better living conditions (higher GDP per capita) are confirmatory. As for the female labor market participation rate, we report a significant and negative association with formal care use. Moreover, although not significant, associations between female labor market participation and informal care are negative, while they are positive for mixed care. Findings of our gender comparisons show first indications that social policies for older adults may be more effective for women than for men (see supplementary material). However, statistically significant gender differences can only be found for the effect of pensions and GDP.

Associations between individual-level and country-level variables and our outcome variables remain consistent when employing a comparatively more subjective indicator like the GALI, which delineates between mild and severe care need. Results, however, reveal that associations between country-level variables and long-term care usage become notably stronger when need is reported to be severe (see Table S.12).

#### Unmet need for personal care

The marginal effect on the uptake of personal care services shown in Table 2 columns 5–7 raises the question of whether observed positive associations with social resources and institutional arrangements also indicate lower levels of unmet need. To draw robust conclusions about disparities in met and unmet need, we rely on the use of ADL—instead of IADL—as upper bound for personal care need. Results of this analysis are reported in Table 3. For better readability and as this table is intended to provide information on unmet need, the formerly polytomous dependent variable is merged into one binary variable distinguishing between care and no care utilization.

**Table 3:** Probability of care need (1 + ADL) and overall utilization – Individual- and country-level average marginal effects

	(Part I) Need for personal care		(Part II) Care use conditional on need		Unconditional care use	
	*(1)* * 1* + *ADL*		*(2) * *care utilization*		*(3)* *care utilization*	
**Individual-level characteristics**						
Socioeconomic status						
*Material resources (ref. 1st wealth quintile)*						
2nd wealth quintile	− 0.026***	(0.006)	− 0.051**	(0.020)	− 0.134***	(0.025)
3rd wealth quintile	− 0.045***	(0.006)	− 0.051**	(0.022)	− 0.213***	(0.030)
4th wealth quintile	− 0.059***	(0.006)	− 0.087***	(0.025)	− 0.304***	(0.033)
5th wealth quintile	− 0.065***	(0.006)	− 0.108***	(0.025)	− 0.353***	(0.031)
*Human resources (ref. primary education)*						
Secondary education	− 0.029***	(0.005)	− 0.037*	(0.019)	− 0.139***	(0.027)
Tertiary education	− 0.056***	(0.006)	− 0.062**	(0.025)	− 0.282***	(0.037)
*Social resources*						
Has spouse/partner	− 0.006*	(0.004)	0.194***	(0.016)	0.149***	(0.019)
Has children	− 0.018***	(0.006)	-0.029	(0.025)	-0.095***	(0.035)
Predisposing factors						
Age	-0.013***	(0.002)	-0.027***	(0.010)	-0.077***	(0.014)
Age^2^	0.00013***	(0.000)	0.00025***	(0.000)	0.00075***	(0.000)
Gender (female)	-0.009**	(0.003)	-0.018	(0.015)	-0.051**	(0.020)
**Country-level characteristics**						
LTC policy						
Means testing (ref. none)	− 0.006	(0.015)	− 0.059	(0.038)	− 0.077***	(0.026)
Cash-for-care benefits (ref. in-kind benefits)	0.025*	(0.013)	0.015	(0.036)	0.119***	(0.023)
LTC beds (p. 1000)	0.001	(0.001)	0.003	(0.002)	0.005***	(0.001)
Pension generosity						
Net replacement rate	− 0.000	(0.000)	0.003***	(0.001)	0.001**	(0.001)
Macroeconomic factors						
Consumer price index (personal care)	0.003*	(0.002)	− 0.004	(0.004)	0.007***	(0.002)
GDP p. capita (in PPS)	− 0.057	(0.035)	− 0.173*	(0.093)	− 0.384***	(0.067)
Female labor market part. (%)	− 0.001	(0.001)	− 0.005	(0.004)	− 0.010***	(0.002)
N	35,547		4246		35,547	

(Bootstrapped) standard errors are in parentheses

*p < 0.10, **p < 0.05, ***p < 0.01

Our findings show that among the individual-level attributes, the presence of a spouse or partner significantly reduces unmet need. More specifically, column (2) reports that having a spouse or partner is associated with a 19.5% increase in the predicted probability of receiving care when there is need. Among the country-specific characteristics, institutional arrangements of the LTC system studied turn out not to have a significant effect on unmet need for personal care. Yet we find that pension generosity measured through higher net replacement rates is positively associated with care use (Table 3).

## Discussion

Previous research suggested that financial and human resources (i.e., wealth and education) are associated with poorer health (Grossman, 1972; Liu & Wang, 2022; Nocera & Zweifel, 1998) but also crucial for the choice of care services when needed (Dong et al., 2021; Ilinca et al., 2017; Lera et al., 2021; Rodrigues et al., 2018). Our findings offer new perspectives by indicating the cumulative effect on older people’s use of home care. In contrast to the preceding literature, we found that wealthier (and better-educated) individuals make less use of personal care even when needs are reported. Descriptive sample statistics reveal that wealthier individuals are not only less likely to need care, but also tend to report milder needs, making them less reliant on personal care and potentially better able to outsource demand to practical household help. In addition, affluent individuals may (better) use advanced technologies such as smart home technologies, creating a better environment, which may allow them to postpone personal care (Korneeva et al., 2021). As wealthier and higher-educated adults might be more able to navigate the sometimes very complex care systems and experience fewer financial barriers to access, they might also be more likely to opt out into institutional care (including day centers and care homes) (Grossman, 1972). However, this potential attrition effect needs to be empirically verified.

Our findings further substantiate the importance of families in their potential to prevent and reduce unmet needs through informal means. However, while children play a greater role in prevention, spouses and partners become more important once there is a need (Ferrer et al., 2005; Pinquart & Sörensen, 2011). Yet results of a gender-specific analysis suggest that the role of the spouse or partner differs significantly between men and women. In particular, men benefit more from informal care when needed, which supports the hypothesis that the highly gendered division of care and housework found in midlife persists when one partner becomes disabled (see supplementary material). According to gender theory, socialization in childhood and reinforcement by norms later in life lead to internalized stable personality traits making men more likely to resist caregiving (Langner & Furstenberg, 2020; Miller & Cafasso, 1992). Moreover, although social resources are likely to play a key role in both preventing and meeting future care needs, possible adverse consequences should not be underestimated, such as potential spillover effects of long-term functional limitations on close family members (Pacheco Barzallo, 2018).

Finally, we fill gaps in the literature by further exploring the role of national policies for older adults, specifically LTC policies. Hereby, our results indicate that countries with fewer restrictions (i.e., the absence of means testing) and more freedom to users to determine the type of care (e.g., Austria, Czech Republic, Finland, Germany and Poland) facilitate access to formal care (see country-level information in Table S.2). However, individuals with functional limitations living in countries providing cash-for-care benefits are more likely to resort to mixed care, that is a combination of formal and informal care. This finding is concordant with the theory of mixed responsibility, which states that welfare state benefits do not lead to a displacement of the family, but to shared responsibility between state and family (Motel-Klingebiel et al., 2005). Our findings align with prior research that underscores the associations between greater state responsibility, reflected in the number of LTC beds, and increased formal care utilization, as it is observed in Northern European countries such as Belgium, Luxembourg, the Netherlands, and Sweden (Floridi et al., 2020; Haberkern & Szydlik, 2010).

Nevertheless, while our findings indicate that the LTC policies examined in this study do not seem to be significantly associated with (lower) unmet personal care needs, they show that countries with generous pension schemes face lower levels of unmet needs through improved access to both informal and formal home care services. The descriptive statistics highlight that specific countries like Austria, Hungary, Italy, Luxembourg, and the Netherlands exhibit notably high net replacement rates, surpassing 80% (see Table S.2). In contrast to existing LTC policies, the generosity of the pension scheme may thus influence both the choice of care (i.e., informal or formal care) as well as the risk of unmet need. In this way, our research most notably contributes to previous findings by shedding light on the potential limits to the effectiveness of existing LTC policies, while strengthening the role of other social interventions for older people (i.e., pensions) in meeting needs.

The macroeconomic associations which show that countries with a higher GDP and lower consumer prices for personal care resort to more formal care are confirmatory. In contrast to Steckenrider (2000), our findings suggest that individuals with functional limitations living in countries with high female labor force participation do not use significantly less informal care. One possible explanation is that the availability of informal caregivers is not significantly reduced, since—as the data show—it is mostly the partner who provides care. Spouses or partners are, however, on average older and may therefore already have left the labor market. Furthermore, as informal care is disproportionately provided by women, our results underline a possible double burden of informal care and work for those still active in the labor market (Stroka & Schmitz, 2012). Moreover, our results show that countries with high female labor force participation are significantly less likely to use formal care exclusively. This may indicate that this double burden could be further exacerbated by a structural shortage of formal caregivers despite high labor force participation (Tarricone & Tsouros, 2008). Finally, it is worth emphasizing—and this does not only apply to the interpretation of the results on female labour force participation—that countries vary greatly in terms of the severity of need. For example, it can be observed that the countries with the highest female labour force participation (i.e., Sweden and Switzerland) are also the countries with the lowest proportion of older adults with severe care need (see Table S.2–S.4).

Although this study provides new insights into the potential drivers of socioeconomic disparities and the role of social policies for older adults in the uptake of informal and formal home care, it is not without limitations. First, interpretations for the total population of older adults should be taken with caution as SHARE is only representative of the population of adults aged 50 and older living in the community (Börsch-Supan et al., 2013). Second, although (I)ADL have a relatively wide scope of application in both empirical literature and policy, it is important to note that these indicators are self-reported and may therefore be subject to bias (Spitzer & Weber, 2019). Third, the concept of (unmet) need is central to our understanding of how welfare states design and provide (LTC) policies for older adults. Using an approach grounded in the literature, we link need with limitations with (I)ADL, which in turn can determine the type of help needed. We conducted a sensitivity analysis by defining need by means of a more subjective indicator (GALI) and found robust results for both individual-level and country-level regressors (see Table S.12). This contrasts with previous findings identifying differences between objective and subjective indicators, with higher utilisation of formal care being typically associated with more subjective indicators (Roquebert et al., 2021). We categorized GALI as a comparatively more subjective indicator compared to ADL and IADL. This categorization arises from its differentiation between mild and severe needs without referencing specific tasks performed. Although it is important to acknowledge that all these measures rely on self-reported data, which introduces the possibility of bias, GALI likely encompasses a wider range of tasks compared to ADL or IADL measures. This broader scope introduces a heightened element of subjectivity for respondents (Tinios & Valvis, 2023). However, considering that our primary measures for need are dichotomous or polytomous in the case of GALI, they might still be limited to comprehensively capture the complexity and severity of need (Tinios & Valvis, 2023; Vlachantoni, 2019). In this way, our two-part multilevel model remains constrained in its comprehensive consideration of the severity of care needs, which would be necessary to ensure reliable conclusions on inequity. Fourth, the cross-sectional nature of our study does not allow us to examine the impact of policy changes over time, which, however, would be a promising pathway for future research. In this study, however, the sampling design of SHARE did not allow us to take this approach without compromising the validity of the results, especially since we would have had to forgo a large number of countries and the associated ability to examine a wide range of social policies for older adults. In particular there have not been fundamental changes in the key indicators of LTC policy over the past decade (i.e., whether cash-for-care benefits are available or means testing is applied), underscoring the strength of the chosen cross-sectional design.

## Conclusion

Building on the theoretical frameworks developed by Grossman (1972) and Andersen and Newman (1973), this study examines the relationship between the utilization of home-based LTC and country-level characteristics in addition to individual-level characteristics across 18 European countries.

First, we conclude that preventive measures designed to reduce socioeconomic disparities may be particularly effective in mitigating potential barriers in accessing informal or formal care services. While wealth and education significantly reduce disparities in need, the presence of social resources, particularly the presence of a spouse or a partner, significantly reduces the likelihood of unmet need through better access to informal care. Evidence that gendered patterns of spousal caregiving persist was found, though, as men have been shown to benefit more from informal care than women.

Second, our results reveal that while existing LTC policies play a key role in the choice of care type (informal or formal), pensions could play a greater role in preventing unmet need. State responsibility for care (through the provision of residential care beds) and other institutional arrangements to support LTC, such as means testing and cash-for-care benefits, are not related to lower unmet need, but merely affect the type of care used (formal, informal or mixed). Nonetheless, the influence on the choice of care type should not be underestimated. Indeed, the aging of the population combined with the increasing participation of women in the labor force may put pressure on families to meet an increasing demand for personal care. While an overall increase in the state’s responsibility and reduction of restrictions in access to services would promote access to formal care, our results suggest that greater availability of cash-for-care benefits would tend to promote mixed forms of care. However, our study stresses that the most potent remedy for unmet personal care needs is pension generosity. Upcoming reforms of pension systems should take into account these possible adverse effects on older people’s access to personal care.

## Supplementary Information

Below is the link to the electronic supplementary material.

Supplementary file1 (DOCX 145 KB)

## Data Availability

The data that support the findings of this study are openly available. Data from OECD and MISSOC (Mutual Information System on Social Protection) are available at https://data.oecd.org/ and https://www.missoc.org/, respectively. Data from the Survey of Health Ageing and Retirement in Europe (SHARE, Wave 8) are available at 10.6103/SHARE.w8.800.
