# Supplementary material for: Informal and formal long-term care utilization and unmet needs in Europe: examining socioeconomic disparities and the role of social policies for older adults
Source: Int J Health Econ Manag. 2024 May 21;25(1):87–106. doi: 10.1007/s10754-024-09378-z (PMC12003520; doi:10.1007/s10754-024-09378-z)
Supplement: Supplementary file 1 — Supplementary file1 (DOCX 145 KB) [file 10754_2024_9378_MOESM1_ESM.docx]

***Supplementary material***

**Informal and Formal Long-Term Care Utilization and Unmet Needs in Europe: Examining Socioeconomic Disparities and the Role of Social Policies for Older Adults**

***Overview***

1. *Descriptives*
2. *Gender differences*
3. *Sensitivity analysis*
4. **Descriptives**

Table S.1: Country sample statistics in total and by care utilization

|  | **no care** | **informal care** | **formal care** | **mixed care** | **Total** |
| --- | --- | --- | --- | --- | --- |
| Austria | 1310 | 50 | 32 | 24 | 1416 |
|  | 92.51 | 3.53 | 2.26 | 1.69 | 100.00 |
| Belgium | 1704 | 59 | 105 | 18 | 1886 |
|  | 90.35 | 3.13 | 5.57 | 0.95 | 100.00 |
| Czech Republic | 2322 | 101 | 20 | 16 | 2459 |
|  | 94.43 | 4.11 | 0.81 | 0.65 | 100.00 |
| Denmark | 2013 | 32 | 45 | 11 | 2101 |
|  | 95.81 | 1.52 | 2.14 | 0.52 | 100.00 |
| Estonia | 2558 | 126 | 23 | 15 | 2722 |
|  | 93.98 | 4.63 | 0.84 | 0.55 | 100.00 |
| Finland | 1032 | 22 | 9 | 0 | 1063 |
|  | 97.08 | 2.07 | 0.85 | 0.00 | 100.00 |
| France | 2239 | 62 | 64 | 18 | 2383 |
|  | 93.96 | 2.60 | 2.69 | 0.76 | 100.00 |
| Germany | 2593 | 70 | 58 | 48 | 2769 |
|  | 93.64 | 2.53 | 2.09 | 1.73 | 100.00 |
| Greece | 2751 | 108 | 32 | 19 | 2910 |
|  | 94.54 | 3.71 | 1.10 | 0.65 | 100.00 |
| Hungary | 672 | 51 | 10 | 2 | 735 |
|  | 91.43 | 6.94 | 1.36 | 0.27 | 100.00 |
| Italy | 1900 | 92 | 24 | 16 | 2032 |
|  | 93.50 | 4.53 | 1.18 | 0.79 | 100.00 |
| Luxembourg | 888 | 10 | 21 | 5 | 924 |
|  | 96.10 | 1.08 | 2.27 | 0.54 | 100.00 |
| Netherlands | 1774 | 31 | 47 | 14 | 1866 |
|  | 95.07 | 1.66 | 2.52 | 0.75 | 100.00 |
| Poland | 1866 | 82 | 12 | 13 | 1973 |
|  | 94.58 | 4.16 | 0.61 | 0.66 | 100.00 |
| Slovenia | 2248 | 107 | 18 | 11 | 2384 |
|  | 94.30 | 4.49 | 0.76 | 0.46 | 100.00 |
| Spain | 1764 | 91 | 72 | 49 | 1976 |
|  | 89.27 | 4.61 | 3.64 | 2.48 | 100.00 |
| Sweden | 2089 | 33 | 47 | 10 | 2179 |
|  | 95.87 | 1.51 | 2.16 | 0.46 | 100.00 |
| Switzerland | 1714 | 12 | 32 | 11 | 1769 |
|  | 96.89 | 0.68 | 1.81 | 0.62 | 100.00 |
| **N** | **33,437** | **1,139** | **671** | **300** | **35,547** |
| **(%)** | **94.06** | **3.20** | **1.89** | **0.84** | **100.00** |
| Note: First row has *frequencies* and second row has *row percentages* | | | | | |
| Table S.2: Country-level information | | | | | |

|  | **Social policies for older adults** | | | | **Macroeconomic indicators** | | |
| --- | --- | --- | --- | --- | --- | --- | --- |
| **Country** | **Cash benefit** | **Means testing** | **LTC beds^1^** | **Pension generosity^2^** | **CPI^3^** | **GDP p. capita** | **FLMP^4^** |
| Austria | Cash | No | 45.9 | 89.9 | 105.1 | 126 | 72.3 |
| Belgium | Cash | Yes | 68.7 | 66.2 | 104.1 | 118 | 64.9 |
| Czech Republic | Cash | No | 35.6 | 60.3 | 110.8 | 93 | 69.8 |
| Denmark | In-kind | No | 37.7 | 70.9 | 95.1 | 127 | 76.0 |
| Estonia | In-kind | Yes | 48.1 | 53.1 | 108.2 | 82 | 75.7 |
| Finland | Cash | No | 54.2 | 64.2 | 101.1 | 109 | 76.6 |
| France | Cash | Yes | 49.1 | 73.6 | 100.9 | 106 | 68.2 |
| Germany | Cash | No | 54.2 | 51.9 | 104.1 | 121 | 74.9 |
| Greece | In-kind | No | 1.8 | 51.1 | 93.1 | 66 | 60.4 |
| Hungary | In-kind | No | 44.5 | 84.3 | 109.4 | 73 | 65.3 |
| Italy | Cash | Yes | 18.8 | 91.8 | 101.4 | 96 | 56.5 |
| Luxembourg | Cash | Yes | 80.8 | 90.1 | 104.7 | 254 | 67.4 |
| Netherlands | Cash | Yes | 72.1 | 80.2 | 106.3 | 128 | 76.7 |
| Poland | Cash | No | 11.3 | 35.1 | 101.5 | 73 | 63.4 |
| Slovenia | Cash | Yes | 51.9 | 57.5 | 101.9 | 88 | 72.2 |
| Spain | Cash | Yes | 43.9 | 83.4 | 99.9 | 91 | 70.1 |
| Sweden | In-kind | No | 68.1 | 53.4 | 109.9 | 119 | 81.1 |
| Switzerland | Cash | Yes | 63.6 | 44.3 | 97.3 | 157 | 80.2 |

p. 1,000 inhabitants aged 65+

^2^ net replacement rate in %

^3^ consumer price index for personal care

^4^ female labor market participation rate in %

Note: Information on cash benefit and means testing are sourced from Ariaans et al (2021) and the Mutual Information System on Social Protection (MISSOC, 2022).

Table S.3 Prevalence of limitations with instrumental activities of daily living (IADL) across countries

| **Country** | **Number of instrumental activities of daily living (IADL)** | | | | | | | | | | |
| --- | --- | --- | --- | --- | --- | --- | --- | --- | --- | --- | --- |
|  | **0** | **1** | **2** | **3** | **4** | **5** | **6** | **7** | **8** | **9** | **Total** |
| Austria | 1,088 | 142 | 58 | 24 | 24 | 23 | 22 | 19 | 7 | 9 | 1,416 |
|  | 76.84 | 10.03 | 4.10 | 1.69 | 1.69 | 1.62 | 1.55 | 1.34 | 0.49 | 0.64 | 100.00 |
| Belgium | 1,442 | 206 | 82 | 55 | 27 | 28 | 18 | 17 | 4 | 7 | 1,886 |
|  | 76.46 | 10.92 | 4.35 | 2.92 | 1.43 | 1.48 | 0.95 | 0.90 | 0.21 | 0.37 | 100.00 |
| Czech Republic | 1,950 | 228 | 84 | 61 | 37 | 30 | 11 | 17 | 16 | 25 | 2,459 |
|  | 79.30 | 9.27 | 3.42 | 2.48 | 1.50 | 1.22 | 0.45 | 0.69 | 0.65 | 1.02 | 100.00 |
| Denmark | 1,797 | 155 | 70 | 23 | 19 | 13 | 13 | 1 | 2 | 8 | 2,101 |
|  | 85.53 | 7.38 | 3.33 | 1.09 | 0.90 | 0.62 | 0.62 | 0.05 | 0.10 | 0.38 | 100.00 |
| Estonia | 1,983 | 296 | 130 | 67 | 67 | 60 | 35 | 40 | 21 | 23 | 2,722 |
|  | 72.85 | 10.87 | 4.78 | 2.46 | 2.46 | 2.20 | 1.29 | 1.47 | 0.77 | 0.84 | 100.00 |
| Finland | 908 | 96 | 25 | 9 | 4 | 6 | 4 | 5 | 2 | 4 | 1,063 |
|  | 85.42 | 9.03 | 2.35 | 0.85 | 0.38 | 0.56 | 0.38 | 0.47 | 0.19 | 0.38 | 100.00 |
| France | 1,909 | 225 | 83 | 57 | 33 | 24 | 18 | 12 | 9 | 13 | 2,383 |
|  | 80.11 | 9.44 | 3.48 | 2.39 | 1.38 | 1.01 | 0.76 | 0.50 | 0.38 | 0.55 | 100.00 |
| Germany | 2,256 | 243 | 66 | 58 | 28 | 28 | 21 | 15 | 14 | 40 | 2,769 |
|  | 81.47 | 8.78 | 2.38 | 2.09 | 1.01 | 1.01 | 0.76 | 0.54 | 0.51 | 1.44 | 100.00 |
| Greece | 2,300 | 248 | 125 | 81 | 48 | 32 | 22 | 10 | 13 | 31 | 2,910 |
|  | 79.04 | 8.52 | 4.30 | 2.78 | 1.65 | 1.10 | 0.76 | 0.34 | 0.45 | 1.07 | 100.00 |
| Hungary | 545 | 67 | 28 | 32 | 24 | 15 | 7 | 10 | 3 | 4 | 735 |
|  | 74.15 | 9.12 | 3.81 | 4.35 | 3.27 | 2.04 | 0.95 | 1.36 | 0.41 | 0.54 | 100.00 |
| Italy | 1,669 | 101 | 63 | 49 | 29 | 31 | 20 | 13 | 17 | 40 | 2,032 |
|  | 82.14 | 4.97 | 3.10 | 2.41 | 1.43 | 1.53 | 0.98 | 0.64 | 0.84 | 1.97 | 100.00 |
| Luxembourg | 812 | 50 | 20 | 12 | 6 | 3 | 4 | 4 | 4 | 9 | 924 |
|  | 87.88 | 5.41 | 2.16 | 1.30 | 0.65 | 0.32 | 0.43 | 0.43 | 0.43 | 0.97 | 100.00 |
| Netherlands | 1,540 | 192 | 48 | 35 | 12 | 16 | 9 | 5 | 4 | 5 | 1,866 |
|  | 82.53 | 10.29 | 2.57 | 1.88 | 0.64 | 0.86 | 0.48 | 0.27 | 0.21 | 0.27 | 100.00 |
| Poland | 1,630 | 103 | 61 | 37 | 24 | 31 | 16 | 19 | 10 | 42 | 1,973 |
|  | 82.62 | 5.22 | 3.09 | 1.88 | 1.22 | 1.57 | 0.81 | 0.96 | 0.51 | 2.13 | 100.00 |
| Slovenia | 1,878 | 198 | 55 | 55 | 41 | 27 | 37 | 25 | 21 | 47 | 2,384 |
|  | 78.78 | 8.31 | 2.31 | 2.31 | 1.72 | 1.13 | 1.55 | 1.05 | 0.88 | 1.97 | 100.00 |
| Spain | 1,506 | 123 | 68 | 40 | 41 | 31 | 37 | 22 | 25 | 83 | 1,976 |
|  | 76.21 | 6.22 | 3.44 | 2.02 | 2.07 | 1.57 | 1.87 | 1.11 | 1.27 | 4.20 | 100.00 |
| Sweden | 1,822 | 180 | 74 | 46 | 14 | 11 | 7 | 13 | 6 | 6 | 2,179 |
|  | 83.62 | 8.26 | 3.40 | 2.11 | 0.64 | 0.50 | 0.32 | 0.60 | 0.28 | 0.28 | 100.00 |
| Switzerland | 1,553 | 120 | 43 | 17 | 16 | 5 | 6 | 3 | 3 | 3 | 1,769 |
|  | 87.79 | 6.78 | 2.43 | 0.96 | 0.90 | 0.28 | 0.34 | 0.17 | 0.17 | 0.17 | 100.00 |
| Total | 28,588 | 2,973 | 1,183 | 758 | 494 | 414 | 307 | 250 | 181 | 399 | 35,547 |
|  | 80.42 | 8.36 | 3.33 | 2.13 | 1.39 | 1.16 | 0.86 | 0.70 | 0.51 | 1.12 | 100.00 |
|  | | | | | | | | | | | |

Table S.4 Prevalence of limitations with activities of daily living (ADL) across countries

| **Country** | **Number of activities of daily living (ADL)** | | | | | | | |  |  |
| --- | --- | --- | --- | --- | --- | --- | --- | --- | --- | --- |
|  | **0** | **1** | **2** | **3** | **4** | **5** | **6** | **Total** |  |  |
| Austria | 1,257 | 75 | 34 | 18 | 9 | 10 | 13 | 1,416 |  |  |
|  | 88.77 | 5.30 | 2.40 | 1.27 | 0.64 | 0.71 | 0.92 | 100.00 |  |  |
| Belgium | 1,578 | 173 | 76 | 26 | 19 | 6 | 8 | 1,886 |  |  |
|  | 83.67 | 9.17 | 4.03 | 1.38 | 1.01 | 0.32 | 0.42 | 100.00 |  |  |
| Czech Republic | 2,095 | 200 | 59 | 46 | 21 | 19 | 19 | 2,459 |  |  |
|  | 85.20 | 8.13 | 2.40 | 1.87 | 0.85 | 0.77 | 0.77 | 100.00 |  |  |
| Denmark | 1,915 | 123 | 33 | 13 | 5 | 7 | 5 | 2,101 |  |  |
|  | 91.15 | 5.85 | 1.57 | 0.62 | 0.24 | 0.33 | 0.24 | 100.00 |  |  |
| Estonia | 2,289 | 183 | 104 | 63 | 37 | 25 | 21 | 2,722 |  |  |
|  | 84.09 | 6.72 | 3.82 | 2.31 | 1.36 | 0.92 | 0.77 | 100.00 |  |  |
| Finland | 956 | 77 | 15 | 8 | 1 | 4 | 2 | 1,063 |  |  |
|  | 89.93 | 7.24 | 1.41 | 0.75 | 0.09 | 0.38 | 0.19 | 100.00 |  |  |
| France | 2,045 | 221 | 52 | 31 | 14 | 12 | 8 | 2383 |  |  |
|  | 85.82 | 9.27 | 2.18 | 1.30 | 0.59 | 0.50 | 0.34 | 100.00 |  |  |
| Germany | 2,407 | 162 | 79 | 43 | 27 | 10 | 41 | 2,769 |  |  |
|  | 86.93 | 5.85 | 2.85 | 1.55 | 0.98 | 0.36 | 1.48 | 100.00 |  |  |
| Greece | 2,711 | 97 | 33 | 17 | 16 | 9 | 27 | 2,910 |  |  |
|  | 93.16 | 3.33 | 1.13 | 0.58 | 0.55 | 0.31 | 0.93 | 100.00 |  |  |
| Hungary | 643 | 47 | 21 | 11 | 2 | 7 | 4 | 735 |  |  |
|  | 87.48 | 6.39 | 2.86 | 1.50 | 0.27 | 0.95 | 0.54 | 100.00 |  |  |
| Italy | 1,795 | 111 | 43 | 21 | 16 | 18 | 28 | 2,032 |  |  |
|  | 88.34 | 5.46 | 2.12 | 1.03 | 0.79 | 0.89 | 1.38 | 100.00 |  |  |
| Luxembourg | 851 | 42 | 9 | 6 | 6 | 7 | 3 | 924 |  |  |
|  | 92.10 | 4.55 | 0.97 | 0.65 | 0.65 | 0.76 | 0.32 | 100.00 |  |  |
| Netherlands | 1,706 | 96 | 33 | 19 | 7 | 2 | 3 | 1,866 |  |  |
|  | 91.43 | 5.14 | 1.77 | 1.02 | 0.38 | 0.11 | 0.16 | 100.00 |  |  |
| Poland | 1,673 | 146 | 57 | 35 | 11 | 19 | 32 | 1,973 |  |  |
|  | 84.79 | 7.40 | 2.89 | 1.77 | 0.56 | 0.96 | 1.62 | 100.00 |  |  |
| Slovenia | 2,109 | 111 | 52 | 34 | 15 | 17 | 46 | 2,384 |  |  |
|  | 88.46 | 4.66 | 2.18 | 1.43 | 0.63 | 0.71 | 1.93 | 100.00 |  |  |
| Spain | 1,658 | 116 | 45 | 36 | 28 | 29 | 64 | 1,976 |  |  |
|  | 83.91 | 5.87 | 2.28 | 1.82 | 1.42 | 1.47 | 3.24 | 100.00 |  |  |
| Sweden | 1,970 | 138 | 41 | 9 | 4 | 9 | 8 | 2,179 |  |  |
|  | 90.41 | 6.33 | 1.88 | 0.41 | 0.18 | 0.41 | 0.37 | 100.00 |  |  |
| Switzerland | 1,643 | 90 | 26 | 2 | 4 | 2 | 2 | 1,769 |  |  |
|  | 92.88 | 5.09 | 1.47 | 0.11 | 0.23 | 0.11 | 0.11 | 100.00 |  |  |
| Total | 31,301 | 2208 | 812 | 438 | 242 | 212 | 334 | 35,547 |  |  |
|  | 88.06 | 6.21 | 2.28 | 1.23 | 0.68 | 0.60 | 0.94 | 100.00 |  |  |
| Note: First row has *frequencies* and second row has *row percentages* | | | | | | | | | | |
|  | | | | | | | | |  |  |

Table S.5: Number of limitations by care utilization among older adults with needs

|  | **no care** | **informal care** | **formal care** | **mixed care** | **Total**  *(in need)* |  |  |
| --- | --- | --- | --- | --- | --- | --- | --- |
| **IADL** |  | | | | |  |  |
| 1 | 2,690 | 195 | 74 | 14 | 2,973 |  |  |
|  | 90.48 | 6.56 | 2.49 | 0.47 | 100.00 |  |  |
| 2 | 977 | 140 | 53 | 13 | 1,183 |  |  |
|  | 82.59 | 11.83 | 4.48 | 1.10 | 100.00 |  |  |
| 3 | 519 | 146 | 73 | 20 | 758 |  |  |
|  | 68.47 | 19.26 | 9.63 | 2.64 | 100.00 |  |  |
| 4 | 319 | 102 | 48 | 25 | 494 |  |  |
|  | 64.57 | 20.65 | 9.72 | 5.06 | 100.00 |  |  |
| 5 | 213 | 95 | 69 | 37 | 414 |  |  |
|  | 51.45 | 22.95 | 16.67 | 8.94 | 100.00 |  |  |
| 6 | 133 | 80 | 59 | 35 | 307 |  |  |
|  | 43.32 | 26.06 | 19.22 | 11.40 | 100.00 |  |  |
| 7 | 92 | 70 | 53 | 35 | 250 |  |  |
|  | 36.80 | 28.00 | 21.20 | 14.00 | 100.00 |  |  |
| 8 | 60 | 61 | 32 | 28 | 181 |  |  |
|  | 33.15 | 33.70 | 17.68 | 15.47 | 100.00 |  |  |
| 9 | 105 | 131 | 76 | 87 | 399 |  |  |
|  | 26.32 | 32.83 | 19.05 | 21.80 | 100.00 |  |  |
| **N** | **5,108** | 1020 | **537** | **294** | **6,959** |  |  |
| **(%)** | **73.40** | 14.66 | **7.72** | **4.22** | **100.00** |  |  |
| **ADL** |  |  |  |  |  |  |  |
| 1 | 1,687 | 317 | 147 | 57 | 2,208 |  |  |
|  | 76.40 | 14.36 | 6.66 | 2.58 | 100.00 |  |  |
| 2 | 440 | 201 | 116 | 55 | 812 |  |  |
|  | 54.19 | 24.75 | 14.29 | 6.77 | 100.00 |  |  |
| 3 | 199 | 134 | 70 | 35 | 438 |  |  |
|  | 45.43 | 30.59 | 15.98 | 7.99 | 100.00 |  |  |
| 4 | 91 | 75 | 43 | 33 | 242 |  |  |
|  | 37.60 | 30.99 | 17.77 | 13.64 | 100.00 |  |  |
| 5 | 58 | 72 | 45 | 37 | 212 |  |  |
|  | 27.36 | 33.96 | 21.23 | 17.45 | 100.00 |  |  |
| 6 | 105 | 96 | 61 | 72 | 334 |  |  |
|  | 31.44 | 28.74 | 18.26 | 21.56 | 100.00 |  |  |
| **N** | **2,580** | 895 | **482** | **289** | **4,246** |  |  |
| **(%)** | **60.76** | 21.08 | **11.35** | **6.81** | **100.00** |  |  |
| Note: First row has *frequencies* and second row has *row percentages* | | | | | | | |

Table S.6: Prevalence of care need (%) by wealth quintile and educational attainment

|  | **Wealth** | | | | | | | |
| --- | --- | --- | --- | --- | --- | --- | --- | --- |
| *IADL* | *1^st^ quintile* | *2^nd^ quintile* | *3^rd^ quintile* | | *4^th^ quintile* | | *5^th^ quintile* | |
| 0 | 18.7 | 17.7 | 18.9 | | 21.2 | | 22.5 | |
| 1 | 26.8 | 20.0 | 21.0 | | 18.2 | | 14.1 | |
| 2 | 28.2 | 30.0 | 13.4 | | 19.3 | | 9.2 | |
| 3 | 37.6 | 25.9 | 16.7 | | 11.1 | | 8.7 | |
| 4 | 41.4 | 24.5 | 16.1 | | 13.0 | | 5.1 | |
| 5 | 50.7 | 11.9 | 13.7 | | 14.5 | | 9.1 | |
| 6 | 40.4 | 19.5 | 14.6 | | 18.1 | | 7.4 | |
| 7 | 45.1 | 22.7 | 15.1 | | 9.3 | | 7.8 | |
| 8 | 37.7 | 24.8 | 14.1 | | 11.9 | | 11.5 | |
| 9 | 32.7 | 27.6 | 22.1 | | 10.4 | | 7.2 | |
|  | **Educational attainment** | | | | | | |  |
| *IADL* | *pre-primary* | | | *secondary* | | *tertiary* | |  |
| 0 | 14.1 | 62.5 | | | 23.5 | | | |
| 1 | 20.0 | 62.9 | | | 17.2 | | | |
| 2 | 31.0 | 58.8 | | | 10.2 | | | |
| 3 | 29.5 | 56.4 | | | 14.1 | | | |
| 4 | 42.8 | 46.6 | | | 10.5 | | | |
| 5 | 34.6 | 57.0 | | | 8.5 | | | |
| 6 | 53.2 | 33.3 | | | 13.4 | | | |
| 7 | 37.9 | 53.4 | | | 8.8 | | | |
| 8 | 49.4 | 43.2 | | | 7.4 | | | |
| 9 | 48.4 | 42.4 | | | 9.3 | | | |

Table S.7: Global activity limitation indicator, in total and by care utilization

|  | (1) | (2) | (3) | (4) | (5) |
| --- | --- | --- | --- | --- | --- |
|  | ***no care*** | ***informal care*** | ***formal care*** | ***mixed care*** | ***Total*** |
|  |  |  |  |  |  |
| *GALI (%)* |  |  |  |  |  |
| no need | 54.7 | 3.3 | 6.6 | 4.0 | 52.2 |
| limited, but not severely | 32.7 | 21.0 | 22.7 | 14.3 | 32.1 |
| severely limited | 12.6 | 75.7 | 70.7 | 81.7 | 15.7 |
| (%) | 100 | 100 | 100 | 100 | 100 |

Note: SHARE data is weighted.

1. **Gender differences**

Table S.8: Probability of care need (1+IADL) and utilization by care type – individual- and country-level average marginal effects (Subsample: Women)

|  |  | (Part I)  **Need for personal care** | (Part II)  **Care use conditional on need** | | | **Unconditional care use** | | |
| --- | --- | --- | --- | --- | --- | --- | --- | --- |
|  |  | *(1)*  *1+ IADL* | *(2)*  *informal care* | *(3)*  *formal care* | *(4)*  *mixed care* | *(5)*  *informal care* | *(6)*  *formal care* | *(7)*  *mixed care* |
| **Individual-level characteristics** | Socioeconomic status |  |  |  |  |  |  |  |
|  | *Material resources*  *(ref. 1^st^ wealth quintile)* |  |  |  |  |  |  |  |
|  | 2^nd^ wealth quintile | -0.030*** (0.008) | -0.045***  (0.013) | -0.008  (0.010) | -0.006  (0.007) | -0.192***  (0.050) | -0.082  (0.051) | -0.102  (0.072) |
|  | 3^rd^ wealth quintile | -0.064*** (0.008) | -0.018  (0.014) | -0.022*  (0.012) | -0.008  (0.008) | -0.133***  (0.048) | -0.166**  (0.069) | -0.128  (0.097) |
|  | 4^th^ wealth quintile | -0.081*** (0.009) | -0.046***  (0.013) | -0.024*  (0.009) | -0.019**  (0.007) | -0.254***  (0.065) | -0.205***  (0.063) | -0.278***  (0.102) |
|  | 5^th^ wealth quintile | -0.106*** (0.010) | -0.015  (0.016) | -0.052***  (0.020) | 0.000  (0.009) | -0.166***  (0.064) | -0.344***  (0.113) | -0.064  (0.122) |
|  |  |  |  |  |  |  |  |  |
|  | *Human resources*  *(ref. primary education)* |  |  |  |  |  |  |  |
|  | secondary education | -0.056*** (0.007) | 0.012  (0.012) | -0.014  (0.009) | -0.006  (0.006) | -0.016  (0.048) | -0.107**  (0.050) | -0.086  (0.073) |
|  | tertiary education | -0.105*** (0.010) | 0.012  (0.017) | -0.029**  (0.015) | -0.010  (0.010) | -0.069  (0.064) | -0.216***  (0.068) | -0.149  (0.111) |
|  |  |  |  |  |  |  |  |  |
|  | *Social resources* |  |  |  |  |  |  |  |
|  | has spouse/partner | -0.003  (0.006) | 0.111***  (0.012) | -0.035***  (0.009) | 0.034 ***  (0.006) | 0.401***  (0.039) | -0.113*  (0.059) | 0.466***  (0.068) |
|  | has children | -0.032*** (0.010) | 0.025  (0.019) | -0.034***  (0.011) | -0.011  (0.011) | 0.053  (0.063) | -0.177***  (0.062) | 0.111  (0.151) |
|  |  |  |  |  |  |  |  |  |
|  | Predisposing factors |  |  |  |  |  |  |  |
|  | age | -0.038*** (0.004) | -0.014**  (0.006) | -0.000  (0.006) | -0.010***  (0.003) | -0.088***  (0.026) | -0.034  (0.034) | -0.120***  (0.040) |
|  | age^2^ | 0.00034*** (0.000) | 0.00011**  (0.000) | 0.00003  (0.000) | 0.00007***  (0.000) | 0.001***  (0.000) | 0.000**  (0.000) | 0.001***  (0.000) |
|  |  |  |  |  |  |  |  |  |
| **Country-level characteristics** |  |  |  |  |  |  |  |  |
|  | LTC policy |  |  |  |  |  |  |  |
|  | means testing (ref. none) | -0.019  (0.018) | -0.002  (0.021) | -0.044***  (0.015) | -0.010  (0.009) | -0.049  (0.050) | -0.253***  (0.063) | -0.147  (0.116) |
|  | cash benefit (ref. in-kind) | -0.017  (0.017) | 0.025  (0.019) | 0.015  (0.014) | 0.028***  (0.006) | 0.100*  (0.055) | 0.099  (0.064) | 0.338***  (0.091) |
|  | LTC beds (p. 1,000) | 0.002** (0.001) | -0.000  (0.001) | 0.003***  (0.001) | -0.000  (0.000) | 0.004*  (0.002) | 0.016***  (0.003) | 0.002  (0.005) |
|  |  |  |  |  |  |  |  |  |
|  | Pension generosity |  |  |  |  |  |  |  |
|  | net replacement rate | 0.001  (0.001) | 0.001  (0.001) | 0.001*  (0.001) | 0.000*  (0.000) | 0.004**  (0.001) | 0.005***  (0.002) | 0.006**  (0.003) |
|  |  |  |  |  |  |  |  |  |
|  | Macroeconomic factors |  |  |  |  |  |  |  |
|  | consumer price index (personal care) | 0.000  (0.002) | 0.002  (0.002) | -0.004**  (0.002) | -0.001  (0.001) | 0.003  (0.005) | -0.019***  (0.007) | -0.017*  (0.010) |
|  | GDP p. capita (in PPS) | -0.142*** (0.042) | -0.215***  (0.058) | 0.012  (0.034) | 0.005  (0.022) | -0.888***  (0.172) | -0.237  (0.153) | -0.116  (0.238) |
|  | female labor market part. (%) | -0.003 (0.002) | -0.002  (0.002) | -0.002*  (0.001) | 0.000  (0.001) | -0.009*  (0.005) | -0.015***  (0.005) | -0.002  (0.009) |
|  | N | 20,165 | 4,526 | | | 20,165 | | |

Note: (Bootstrapped) standard errors are in parentheses ^*^ *p* < 0.10, ^**^ *p* < 0.05, ^***^ *p* < 0.01

Table S.9: Probability of care need (1+IADL) and utilization by care type – individual- and country-level average marginal effects (Subsample: Men)

|  |  | (Part I)  **Need for personal care** | (Part II)  **Care use conditional on need** | | | **Unconditional care use** | | |
| --- | --- | --- | --- | --- | --- | --- | --- | --- |
|  |  | *(1)*  *1+ IADL* | *(2)*  *informal care* | *(3)*  *formal care* | *(4)*  *mixed care* | *(5)*  *informal care* | *(6)*  *formal care* | *(7)*  *mixed care* |
| **Individual-level characteristics** | Socioeconomic status |  |  |  |  |  |  |  |
|  | *Material resources*  *(ref. 1^st^ wealth quintile)* |  |  |  |  |  |  |  |
|  | 2^nd^ wealth quintile | -0.024^***^ (0.008) | 0.006  (0.022) | -0.026**  (0.013) | -0.025*  (0.013) | -0.050  (0.042) | -0.149^**^  (0.062) | -0.154^**^  (0.070) |
|  | 3^rd^ wealth quintile | -0.066^***^ (0.009) | -0.004  (0.024) | -0.003  (0.014) | -0.012  (0.014) | -0.126^***^  (0.042) | -0.059  (0.063) | -0.101  (0.064) |
|  | 4^th^ wealth quintile | -0.063^***^ (0.009) | -0.006  (0.024) | -0.025  (0.015) | -0.022  (0.014) | -0.139^***^  (0.049) | -0.169^**^  (0.081) | -0.161^*^  (0.083) |
|  | 5^th^ wealth quintile | -0.089^***^ (0.010) | -0.042  (0.028) | -0.061***  (0.021) | -0.021  (0.016) | -0.264^***^  (0.055) | -0.371^***^  (0.105) | -0.204^**^  (0.082) |
|  |  |  |  |  |  |  |  |  |
|  | *Human resources*  *(ref. primary education)* |  |  |  |  |  |  |  |
|  | secondary education | -0.049*** (0.008) | 0.001  (0.020) | -0.001  (0.012) | -0.006  (0.012) | -0.087**  (0.044) | -0.037  (0.066) | -0.058  (0.068) |
|  | tertiary education | -0.099*** (0.010) | 0.005  (0.027) | -0.011  (0.015) | -0.007  (0.015) | -0.179***  (0.052) | -0.015  (0.073) | -0.085  (0.072) |
|  |  |  |  |  |  |  |  |  |
|  | *Social resources* |  |  |  |  |  |  |  |
|  | has spouse/partner | -0.014** (0.007) | 0.185***  (0.024) | -0.044***  (0.010) | 0.056***  (0.015) | 0.343***  (0.050) | -0.130**  (0.052) | 0.358***  (0.078) |
|  | has children | -0.038*** (0.009) | 0.029  (0.030) | -0.040***  (0.013) | -0.019  (0.016) | -0.031  (0.056) | -0.214***  (0.057) | -0.123  (0.082) |
|  |  |  |  |  |  |  |  |  |
|  | Predisposing factors |  |  |  |  |  |  |  |
|  | age | -0.026*** (0.005) | 0.033**  (0.013) | -0.009  (0.007) | 0.001  (0.008) | 0.014  (0.025) | -0.049  (0.032) | 0.003  (0.034) |
|  | age^2^ | 0.00024 *** (0.000) | -0.00021**  (0.000) | 0.00001*  (0.000) | 0.00001  (0.000) | 0.000  (0.000) | 0.000**  (0.000) | 0.000  (0.000) |
|  |  |  |  |  |  |  |  |  |
| **Country-level characteristics** |  |  |  |  |  |  |  |  |
|  | LTC policy |  |  |  |  |  |  |  |
|  | means testing (ref. none) | -0.019 (0.018) | 0.015  (0.028) | -0.020  (0.017) | -0.017  (0.015) | -0.019  (0.044) | -0.111  (0.077) | -0.101  (0.070) |
|  | cash benefit (ref. in-kind) | 0.015  (0.017) | 0.004  (0.027) | -0.014  (0.015) | 0.022*  (0.014) | 0.040  (0.046) | -0.044  (0.074) | 0.122*  (0.066) |
|  | LTC beds (p. 1,000) | 0.001 (0.001) | -0.001  (0.001) | 0.002**  (0.001) | -0.000  (0.001) | 0.000  (0.002) | 0.010***  (0.003) | -0.000  (0.004) |
|  |  |  |  |  |  |  |  |  |
|  | Pension generosity |  |  |  |  |  |  |  |
|  | net replacement rate | -0.002 (0.001) | 0.001  (0.000) | 0.000  (0.000) | 0.000  (0.000) | 0.001  (0.001) | 0.002  (0.002) | 0.001  (0.002) |
|  |  |  |  |  |  |  |  |  |
|  | Macroeconomic factors |  |  |  |  |  |  |  |
|  | consumer price index (personal care) | 0.003  (0.002) | 0.010***  (0.003) | -0.005***  (0.002) | -0.001  (0.001) | 0.021***  (0.005) | -0.021***  (0.008) | -0.002  (0.008) |
|  | GDP p. capita (in PPS) | -0.103** (0.041) | -0.157***  (0.079) | 0.047  (0.036) | 0.006  (0.040) | -0.461***  (0.137) | 0.110  (0.170) | -0.073  (0.179) |
|  | female labor market part. (%) | -0.002 (0.002) | -0.004  (0.003) | -0.004***  (0.001) | 0.003*  (0.002) | -0.010**  (0.005) | -0.018***  (0.005) | 0.010  (0.007) |
|  | N | 15,382 | 2,433 | | | 15,382 | | |

Note: (Bootstrapped) standard errors are in parentheses ^*^ *p* < 0.10, ^**^ *p* < 0.05, ^***^ *p* < 0.01

Table S.10: Probability of care need (1+ADL) and overall care utilization – individual- and country-level average marginal effects (Subsample: Women)

|  |  | (Part I)  **Need for personal care** | | (Part II)  **Care use conditional on need** | | **Unconditional care use** | |
| --- | --- | --- | --- | --- | --- | --- | --- |
|  |  | *1+ADL* | | *(2)*  *care utilization* | | *(3)*  *care utilization* | |
| **Individual-level characteristics** | Socioeconomic status |  |  |  |  |  |  |
|  | *Material resources*  *(ref. 1^st^ wealth quintile)* |  |  |  |  |  |  |
|  | 2nd wealth quintile | -0.019*** | (0.006) | -0.062** | (0.025) | -0.136*** | (0.036) |
|  | 3rd wealth quintile | -0.034*** | (0.007) | -0.070** | (0.028) | -0.200*** | (0.039) |
|  | 4th wealth quintile | -0.051*** | (0.007) | -0.119*** | (0.031) | -0.318*** | (0.047) |
|  | 5th wealth quintile | -0.056*** | (0.008) | -0.064* | (0.035) | -0.291*** | (0.053) |
|  |  |  |  |  |  |  |  |
|  | *Human resources*  *(ref. primary education)* |  |  |  |  |  |  |
|  | secondary education | -0.024*** | (0.006) | -0.045* | (0.024) | -0.139*** | (0.033) |
|  | tertiary education | -0.054*** | (0.008) | -0.089*** | (0.034) | -0.299*** | (0.045) |
|  |  |  |  |  |  |  |  |
|  | *Social resources* |  |  |  |  |  |  |
|  | has spouse/partner | -0.009* | (0.005) | 0.193*** | (0.021) | 0.151*** | (0.032) |
|  | has children | -0.018** | (0.008) | -0.011 | (0.032) | -0.080* | (0.043) |
|  |  |  |  |  |  |  |  |
|  | Predisposing factors |  |  |  |  |  |  |
|  | age | -0.017*** | (0.003) | -0.042*** | (0.012) | -0.107*** | (0.017) |
|  | age^2^ | 0.00016*** | (0.000) | 0.00035*** | (0.000) | 0.001*** | (0.000) |
| **Country-level characteristics** |  |  |  |  |  |  |  |
|  | LTC policy |  |  |  |  |  |  |
|  | means testing (ref. none) | -0.008 | (0.016) | -0.089** | (0.035) | -0.117*** | (0.036) |
|  | cash-for-care benefits (ref. in-kind) | 0.020 | (0.014) | 0.038 | (0.033) | 0.118*** | (0.036) |
|  | LTC beds (p. 1,000) | 0.001 | (0.001) | 0.005** | (0.002) | 0.007*** | (0.002) |
|  |  |  |  |  |  |  |  |
|  | Pension generosity |  |  |  |  |  |  |
|  | net replacement rate | -0.000 | (0.000) | 0.003*** | (0.001) | 0.003*** | (0.001) |
|  |  |  |  |  |  |  |  |
|  | Macroeconomic factors |  |  |  |  |  |  |
|  | consumer price index (personal care) | 0.002 | (0.002) | -0.008** | (0.004) | 0.001 | (0.003) |
|  | GDP p. capita (in PPS) | -0.071* | (0.037) | -0.206** | (0.089) | -0.478*** | (0.101) |
|  | female labor market part. (%) | -0.002 | (0.002) | -0.003 | (0.003) | -0.012*** | (0.004) |
|  | N | 20,165 | | 2,414 | | 20165 | |

Note: (Bootstrapped) standard errors are in parentheses ^*^ *p* < 0.10, ^**^ *p* < 0.05, ^***^ *p* < 0.01

Table S.11: Probability of care need (1+ADL) and overall care utilization – individual- and country-level average marginal effects (Subsample: Men)

|  |  | (Part I)  **Need for personal care** | | (Part II)  **Care use conditional on need** | | **Unconditional care use** | |
| --- | --- | --- | --- | --- | --- | --- | --- |
|  |  | *1+ADL* | | *(2)*  *care utilization* | | *(3)*  *care utilization* | |
| **Individual-level characteristics** | Socioeconomic status |  |  |  |  |  |  |
|  | *Material resources*  *(ref. 1^st^ wealth quintile)* |  |  |  |  |  |  |
|  | 2^nd^ wealth quintile | -0.027*** | (0.007) | -0.030 | (0.031) | -0.137*** | (0.040) |
|  | 3^rd^ wealth quintile | -0.052*** | (0.008) | -0.020 | (0.033) | -0.239*** | (0.043) |
|  | 4^th^ wealth quintile | -0.063*** | (0.008) | -0.051 | (0.035) | -0.308*** | (0.054) |
|  | 5^th^ wealth quintile | -0.071*** | (0.009) | -0.156*** | (0.038) | -0.427*** | (0.051) |
|  |  |  |  |  |  |  |  |
|  | *Human resources*  *(ref. primary education)* |  |  |  |  |  |  |
|  | secondary education | -0.027*** | (0.007) | -0.017 | (0.030) | -0.128*** | (0.042) |
|  | tertiary education | -0.055*** | (0.009) | -0.027 | (0.037) | -0.255*** | (0.048) |
|  |  |  |  |  |  |  |  |
|  | *Social resources* |  |  |  |  |  |  |
|  | has spouse/partner | -0.001 | (0.006) | 0.192*** | (0.027) | 0.149*** | (0.040) |
|  | has children | -0.015* | (0.009) | -0.062 | (0.039) | -0.114*** | (0.043) |
|  |  |  |  |  |  |  |  |
|  | Predisposing factors |  |  |  |  |  |  |
|  | age | -0.008^*^ | (0.004) | -0.005 | (0.018) | -0.029 | (0.022) |
|  | age^2^ | 0.00009^***^ | (0.000) | 0.00004 | (0.000) | 0.000*** | (0.000) |
| **Country-level characteristics** |  |  |  |  |  |  |  |
|  | LTC policy |  |  |  |  |  |  |
|  | means testing (ref. none) | -0.001 | (0.016) | -0.041 | (0.049) | -0.038 | (0.044) |
|  | cash-for-care benefits (ref. in-kind) | 0.034** | (0.016) | -0.012 | (0.046) | 0.135*** | (0.043) |
|  | LTC beds (p. 1,000) | 0.000 | (0.000) | 0.003 | (0.002) | 0.002 | (0.002) |
|  |  |  |  |  |  |  |  |
|  | Pension generosity |  |  |  |  |  |  |
|  | net replacement rate | -0.001 | (0.000) | 0.003** | (0.001) | -0.001 | (0.001) |
|  |  |  |  |  |  |  |  |
|  | Macroeconomic factors |  |  |  |  |  |  |
|  | consumer price index (personal care) | 0.004** | (0.002) | -0.003 | (0.005) | 0.013*** | (0.004) |
|  | GDP p. capita (in PPS) | -0.033 | (0.038) | -0.174 | (0.119) | -0.276** | (0.113) |
|  | female labor market part. (%) | -0.000 | (0.002) | -0.006 | (0.004) | -0.006 | (0.005) |
|  | N | 15,382 | | 1,832 | | 15,382 | |

Note: (Bootstrapped) standard errors are in parentheses ^*^ *p* < 0.10, ^**^ *p* < 0.05, ^***^ *p* < 0.01

1. **Sensitivity analysis**^[[1]](#footnote-1)^

Table S.12: Probability of care need (*GALI*) and utilization by care type – individual- and country-level average marginal effects

|  |  | (Part I)  **Need for personal care** | | (Part II)  **Care use conditional on need** | | | | | |
| --- | --- | --- | --- | --- | --- | --- | --- | --- | --- |
|  |  |  | | ***mild need*** | | | ***severe need*** | | |
|  |  | *(1)*  *mild need* | *(2)*  *severe need* | *(3)*  *informal care* | *(4)*  *formal care* | *(5)*  *mixed care* | *(6)*  *informal care* | *(7)*  *formal care* | *(8)*  *mixed care* |
| **Individual-level characteristics** | Socioeconomic status |  |  |  |  |  |  |  |  |
|  | *Material resources*  *(ref. 1^st^ wealth quintile)* |  |  |  |  |  |  |  |  |
|  | 2^nd^ wealth quintile | -0.007  (0.008) | -0.042^***^  (0.006) | -0.008  (0.005) | -0.004  (0.003) | -0.002  (0.002) | -0.018  (0.013) | -0.019^**^  (0.009) | -0.009  (0.007) |
|  | 3^rd^ wealth quintile | -0.014^*^  (0.008) | -0.068^***^  (0.006) | -0.009^*^  (0.005) | -0.006^*^  (0.003) | -0.003  (0.002) | -0.016  (0.013) | -0.016  (0.010) | -0.005  (0.008) |
|  | 4^th^ wealth quintile | -0.036^***^  (0.008) | -0.082^***^  (0.007) | -0.011^**^  (0.005) | -0.006^*^  (0.003) | -0.005^***^  (0.002) | -0.025^*^  (0.014) | -0.027^***^  (0.010) | -0.008  (0.008) |
|  | 5^th^ wealth quintile | -0.038^***^  (0.008) | -0.092^***^  (0.007) | -0.006  (0.005) | -0.006  (0.004) | -0.003  (0.002) | -0.043^***^  (0.014) | -0.047^***^  (0.011) | -0.005  (0.009) |
|  |  |  |  |  |  |  |  |  |  |
|  | *Human resources*  *(ref. primary education)* |  |  |  |  |  |  |  |  |
|  | secondary education | -0.030^***^  (0.008) | -0.028^***^  (0.006) | -0.003  (0.004) | -0.003  (0.003) | -0.003^**^  (0.002) | -0.005  (0.012) | -0.018^**^  (0.008) | -0.009  (0.007) |
|  | tertiary education | -0.044^***^  (0.009) | -0.068^***^  (0.007) | -0.004  (0.005) | -0.003  (0.003) | -0.003  (0.002) | -0.015  (0.015) | -0.018  (0.011) | -0.014  (0.008) |
|  |  |  |  |  |  |  |  |  |  |
|  | *Social resources* |  |  |  |  |  |  |  |  |
|  | has spouse/partner | 0.001  (0.006) | 0.002  (0.004) | 0.020^***^  (0.003) | -0.012^***^  (0.003) | 0.003^**^  (0.001) | 0.119^***^  (0.010) | -0.033^***^  (0.008) | 0.040^***^  (0.005) |
|  | has children | -0.001  (0.009) | -0.029^***^  (0.007) | 0.008^*^  (0.005) | -0.007^*^  (0.004) | -0.004  (0.003) | 0.003  (0.016) | -0.035^***^  (0.012) | 0.008  (0.009) |
|  |  |  |  |  |  |  |  |  |  |
|  | Predisposing factors |  |  |  |  |  |  |  |  |
|  | age | 0.004^***^  (0.001) | 0.000  (0.001) | 0.001^**^  (0.000) | 0.000  (0.000) | 0.000  (0.000) | 0.003^**^  (0.001) | 0.003^***^  (0.001) | 0.001  (0.001) |
|  | age^2^ | 0.000^**^  (0.000) | 0.000^**^  (0.000) | 0.000  (0.000) | 0.000^***^  (0.000) | 0.000^***^  (0.000) | 0.000  (0.000) | 0.000  (0.000) | 0.000  (0.000) |
|  | gender (female) | 0.033^***^  (0.005) | -0.003  (0.004) | -0.001  (0.003) | -0.000  (0.002) | -0.002  (0.001) | -0.023^**^  (0.009) | 0.005  (0.007) | 0.000  (0.005) |
|  |  |  |  |  |  |  |  |  |  |
| **Country-level characteristics** |  |  |  |  |  |  |  |  |  |
|  | LTC policy |  |  |  |  |  |  |  |  |
|  | means testing (ref. none) | -0.021  (0.025) | 0.039^***^  (0.012) | -0.009^*^  (0.005) | -0.010^*^  (0.005) | -0.005  (0.004) | -0.016  (0.021) | -0.038^***^  (0.014) | -0.015  (0.010) |
|  | cash benefit (ref. in-kind) | 0.041^*^  (0.024) | 0.015  (0.012) | 0.001  (0.005) | -0.003  (0.004) | 0.003^**^  (0.001) | -0.002  (0.021) | -0.004  (0.013) | 0.014^**^  (0.007) |
|  | LTC beds (p. 1,000) | -0.000  (0.001) | -0.000  (0.001) | 0.000  (0.000) | 0.001^***^  (0.000) | 0.000^*^  (0.000) | 0.001  (0.001) | 0.003^***^  (0.001) | -0.000  (0.000) |
|  |  |  |  |  |  |  |  |  |  |
|  | Pension generosity |  |  |  |  |  |  |  |  |
|  | net replacement rate | -0.000  (0.001) | -0.001^**^  (0.000) | 0.000  (0.000) | 0.000  (0.000) | 0.000^*^  (0.000) | 0.002^***^  (0.001) | 0.001^***^  (0.000) | 0.000^*^  (0.000) |
|  |  |  |  |  |  |  |  |  |  |
|  | Macroeconomic factors |  |  |  |  |  |  |  |  |
|  | consumer price index (personal care) | 0.004  (0.003) | 0.009^***^  (0.001) | -0.001^*^  (0.001) | -0.002^***^  (0.000) | -0.000  (0.000) | 0.001  (0.002) | -0.006^***^  (0.001) | -0.002^**^  (0.001) |
|  | GDP p. capita (in PPS) | -0.002  (0.058) | -0.008  (0.029) | -0.049^***^  (0.016) | -0.014^*^  (0.008) | -0.010^**^  (0.005) | -0.249^***^  (0.057) | -0.021  (0.030) | 0.005  (0.023) |
|  | female labor market part. (%) | 0.002  (0.002) | 0.001  (0.001) | -0.000  (0.001) | -0.001^***^  (0.000) | -0.000  (0.000) | -0.003  (0.002) | -0.002^**^  (0.001) | 0.001  (0.001) |
|  | N | 35,547 | | 11,544 | | | 5,927 | | |

Note: Standard errors are in parentheses ^*^ *p* < 0.10, ^**^ *p* < 0.05, ^***^ *p* < 0.01

Table S.13: Probability of using informal, formal or mixed care – individual- and country-level average marginal effects (*GALI*)

|  |  | **Unconditional care use** | | |
| --- | --- | --- | --- | --- |
|  |  | *(1)*  *informal care* | *(2)*  *formal care* | *(3)*  *mixed care* |
| **Individual-level characteristics** | Socioeconomic status |  |  |  |
|  | *Material resources*  *(ref. 1^st^ wealth quintile)* |  |  |  |
|  | 2^nd^ wealth quintile | -0.158^***^  (0.057) | -0.160^**^  (0.075) | -0.213  (0.172) |
|  | 3^rd^ wealth quintile | -0.187^***^  (0.062) | -0.229^***^  (0.088) | -0.293^*^  (0.173) |
|  | 4^th^ wealth quintile | -0.247^***^  (0.067) | -0.266^***^  (0.096) | -0.749  (2.136) |
|  | 5^th^ wealth quintile | -0.216^***^  (0.067) | -0.338^***^  (0.120) | -0.303  (0.226) |
|  |  |  |  |  |
|  | *Human resources*  *(ref. primary education)* |  |  |  |
|  | secondary education | -0.077  (0.055) | -0.138^*^  (0.075) | -0.363^**^  (0.168) |
|  | tertiary education | -0.136^*^  (0.076) | -0.158  (0.097) | -0.357  (0.221) |
|  |  |  |  |  |
|  | *Social resources* |  |  |  |
|  | has spouse/partner | 0.556^***^  (0.055) | -0.343^***^  (0.070) | 0.518^***^  (0.180) |
|  | has children | 0.119  (0.116) | -0.251^***^  (0.096) | -0.248  (0.161) |
|  |  |  |  |  |
|  | Predisposing factors |  |  |  |
|  | gender (female) | -0.044  (0.049) | 0.007  (0.062) | -0.157  (0.142) |
|  | age | 0.025^***^  (0.007) | 0.014  (0.011) | 0.012  (0.026) |
|  | age^2^ | 0.000  (0.000) | 0.001^***^  (0.000) | 0.001^**^  (0.001) |
|  |  |  |  |  |
| **Country-level characteristics** |  |  |  |  |
|  | LTC policy |  |  |  |
|  | means testing (ref. none) | -0.176^***^  (0.063) | -0.356^***^  (0.102) | -0.513^***^  (0.151) |
|  | cash benefit (ref. in-kind) | 0.035  (0.062) | -0.064  (0.087) | 0.408^*^  (0.216) |
|  | LTC beds (p. 1,000) | 0.006^**^  (0.003) | 0.034^***^  (0.005) | 0.018^**^  (0.008) |
|  |  |  |  |  |
|  | Pension generosity |  |  |  |
|  | net replacement rate | 0.005^***^  (0.002) | 0.007^***^  (0.002) | 0.011^***^  (0.004) |
|  |  |  |  |  |
|  | Macroeconomic factors |  |  |  |
|  | consumer price index (personal care) | -0.013^**^  (0.006) | -0.065^***^  (0.010) | -0.041^**^  (0.019) |
|  | GDP p. capita (in PPS) | -1.151^***^  (0.191) | -0.537^***^  (0.202) | -1.082^***^  (0.403) |
|  | female labor market part. (%) | -0.011^*^  (0.007) | -0.031^***^  (0.007) | -0.016  (0.018) |
|  | N | 35,547 | | |

Note: (Bootstrapped) standard errors are in parentheses ^*^ *p* < 0.10, ^**^ *p* < 0.05, ^***^ *p* < 0.01

Table S.14: Probability of care need (1+IADL) and utilization by care type – individual- and country-level average marginal effects (including financial resources)

|  |  | (Part I)  **Need for personal care** | (Part II)  **Care use conditional on need** | | | **Unconditional care use** | | |
| --- | --- | --- | --- | --- | --- | --- | --- | --- |
|  |  | *(1)*  *1+ IADL* | *(2)*  *informal care* | *(3)*  *formal care* | *(4)*  *mixed care* | *(5)*  *informal care* | *(6)*  *formal care* | *(7)*  *mixed care* |
| **Individual-level characteristics** | Socioeconomic status |  |  |  |  |  |  |  |
|  | *Financial resources*  *(ref. 1^st^ income quintile)* |  |  |  |  |  |  |  |
|  | 2nd income quintile | -0.020^***^ (0.006) | 0.016  (0.012) | -0.014^*^  (0.008) | 0.006  (0.006) | 0.025  (0.032) | -0.068^*^  (0.037) | 0.054  (0.064) |
|  | 3rd income quintile | -0.025^***^  (0.007) | 0.018  (0.013) | -0.016^*^  (0.010) | 0.013^*^  (0.007) | 0.026  (0.033) | -0.077  (0.054) | 0.100  (0.064) |
|  | 4th income quintile | -0.038^***^  (0.007) | 0.046^***^  (0.015) | -0.035^***^  (0.011) | 0.012  (0.008) | 0.078^*^  (0.042) | -0.184^***^  (0.068) | 0.093  (0.077) |
|  | 5th income quintile | -0.063^***^  (0.007) | 0.029^*^  (0.016) | -0.036^***^  (0.012) | 0.004  (0.009) | 0.001  (0.047) | -0.214^***^  (0.080) | 0.015  (0.082) |
|  | home ownership (ref. no) | -0.035^***^  (0.005) | -0.028^***^  (0.010) | -0.023^***^  (0.007) | -0.002  (0.006) | -0.125^***^  (0.028) | -0.146^***^  (0.034) | -0.057  (0.048) |
|  | *Human resources*  *(ref. primary education)* |  |  |  |  |  |  |  |
|  | secondary education | -0.068^***^  (0.007) | 0.001  (0.011) | -0.010  (0.007) | -0.010  (0.006) | -0.073^**^  (0.033) | -0.091^**^  (0.037) | -0.107^**^  (0.051) |
|  | tertiary education | -0.116^***^  (0.008) | -0.009  (0.014) | -0.014  (0.010) | -0.013^*^  (0.008) | -0.164^***^  (0.044) | -0.147^***^  (0.055) | -0.160^**^  (0.077) |
|  | *Social resources* |  |  |  |  |  |  |  |
|  | has spouse/partner | -0.003 (0.005) | 0.112^***^  (0.010) | -0.031^***^  (0.007) | 0.034^***^  (0.005) | 0.338^***^  (0.029) | -0.092^**^  (0.036) | 0.351^***^  (0.058) |
|  | has children | -0.038^***^ (0.008) | 0.027^*^  (0.014) | -0.041^***^  (0.011) | -0.000  (0.009) | 0.023  (0.050) | -0.188^***^  (0.049) | -0.018  (0.074) |
|  |  |  |  |  |  |  |  |  |
|  | Predisposing factors |  |  |  |  |  |  |  |
|  | age | 0.002*** (0.001) | 0.001  (0.001) | 0.002^**^  (0.001) | -0.000  (0.001) | 0.006  (0.004) | 0.014^**^  (0.006) | 0.000  (0.007) |
|  | age^2^ | 0.000*** (0.000) | 0.000  (0.000) | 0.000^*^  (0.000) | 0.000^**^  (0.000) | 0.000^***^  (0.000) | 0.000^***^  (0.000) | 0.001^***^  (0.000) |
|  | gender (female) | 0.051*** (0.004) | -0.044^***^  (0.009) | -0.003  (0.007) | -0.011^**^  (0.005) | -0.070^***^  (0.026) | -0.008  (0.032) | -0.101^**^  (0.040) |
|  |  |  |  |  |  |  |  |  |
| **Country-level characteristics** |  |  |  |  |  |  |  |  |
|  | LTC policy |  |  |  |  |  |  |  |
|  | means testing (ref. none) | -0.018  (0.017) | 0.006  (0.018) | -0.033^***^  (0.012) | -0.010  (0.008) | -0.023  (0.032) | -0.174^***^  (0.049) | -0.105^**^  (0.053) |
|  | cash-for-care benefit (ref. in-kind benefits) | -0.006  (0.016) | 0.014  (0.017) | 0.000  (0.011) | 0.021^***^  (0.006) | 0.046  (0.035) | 0.015  (0.042) | 0.208^***^  (0.054) |
|  | LTC beds (p. 1,000) | 0.002^**^  (0.001) | -0.000  (0.001) | 0.002^***^  (0.001) | -0.000  (0.000) | 0.003  (0.002) | 0.014^***^  (0.002) | 0.001  (0.003) |
|  |  |  |  |  |  |  |  |  |
|  | Pension generosity |  |  |  |  |  |  |  |
|  | net replacement rate | 0.000  (0.000) | 0.001  (0.000) | 0.001^**^  (0.000) | 0.000  (0.000) | 0.003^***^  (0.001) | 0.004^***^  (0.001) | 0.003^**^  (0.001) |
|  |  |  |  |  |  |  |  |  |
|  | Macroeconomic factors |  |  |  |  |  |  |  |
|  | consumer price index(personal care) | 0.001  (0.002) | 0.004^**^  (0.002) | -0.004^***^  (0.001) | -0.001  (0.001) | 0.010^***^  (0.003) | -0.020^***^  (0.005) | -0.008  (0.006) |
|  | GDP p. capita (in PPS) | -0.134^***^  (0.039) | -0.198^***^  (0.050) | 0.009  (0.026) | 0.009  (0.020) | -0.695^***^  (0.130) | -0.107  (0.117) | -0.074  (0.118) |
|  | female labor market part. (%) | -0.003^*^ (0.002) | -0.002  (0.002) | -0.003^***^  (0.001) | 0.001  (0.001) | -0.011^***^  (0.004) | -0.019^***^  (0.004) | 0.004  (0.005) |
|  | N | 35,547 | 6,959 | | | 35,547 | | |

Note: (Bootstrapped) standard errors are in parentheses ^*^ *p* < 0.10, ^**^ *p* < 0.05, ^***^ *p* < 0.01

Table S.15: Probability of care need (1+IADL) and utilization by care type – individual- and country-level average marginal effects (excluding wealth)

|  |  | (Part I)  **Need for personal care** | (Part II)  **Care use conditional on need** | | | **Unconditional care use** | | |
| --- | --- | --- | --- | --- | --- | --- | --- | --- |
|  |  | *(1)*  *1+ IADL* | *(2)*  *informal care* | *(3)*  *formal care* | *(4)*  *mixed care* | *(5)*  *informal care* | *(6)*  *formal care* | *(7)*  *mixed care* |
|  |  |  |  |  |  |  |  |  |
| **Individual-level characteristics** | Socioeconomic status |  |  |  |  |  |  |  |
|  | *Human resources*  *(ref. primary education)* |  |  |  |  |  |  |  |
|  | secondary education | -0.077^***^  (0.007) | 0.004  (0.011) | -0.014^*^  (0.007) | -0.009  (0.006) | -0.075^**^  (0.033) | -0.111^***^  (0.037) | -0.105^**^  (0.049) |
|  | tertiary education | -0.134^***^  (0.008) | -0.003  (0.014) | -0.023^**^  (0.010) | -0.013  (0.008) | -0.169^***^  (0.043) | -0.200^***^  (0.053) | -0.158^**^  (0.071) |
|  | *Social resources* |  |  |  |  |  |  |  |
|  | has spouse/partner | -0.023^***^  (0.004) | 0.120^***^  (0.009) | -0.045^***^  (0.007) | 0.037^***^  (0.005) | 0.332^***^  (0.027) | -0.164^***^  (0.032) | 0.366^***^  (0.053) |
|  | has children | -0.039^***^ (0.008) | 0.027^*^  (0.014) | -0.041^***^  (0.011) | 0.000  (0.009) | 0.021  (0.050) | -0.187^***^  (0.048) | -0.018  (0.073) |
|  |  |  |  |  |  |  |  |  |
|  | Predisposing factors |  |  |  |  |  |  |  |
|  | age | 0.002^***^ (0.001) | 0.001  (0.001) | 0.002^**^  (0.001) | -0.000  (0.001) | 0.006  (0.004) | 0.014^**^  (0.006) | 0.001  (0.007) |
|  | age^2^ | 0.000^***^ (0.000) | 0.000  (0.000) | 0.000^*^  (0.000) | 0.000^**^  (0.000) | 0.000^***^  (0.000) | 0.000^***^  (0.000) | 0.001^***^  (0.000) |
|  | gender (female) | 0.053^***^ (0.004) | -0.046^***^  (0.009) | -0.002  (0.007) | -0.012^**^  (0.005) | -0.070^***^  (0.026) | -0.002  (0.031) | -0.103^***^  (0.039) |
|  |  |  |  |  |  |  |  |  |
| **Country-level characteristics** |  |  |  |  |  |  |  |  |
|  | LTC policy |  |  |  |  |  |  |  |
|  | means testing (ref. none) | -0.023  (0.017) | 0.004  (0.018) | -0.038^***^  (0.013) | -0.010  (0.008) | -0.034  (0.032) | -0.200^***^  (0.048) | -0.105^**^  (0.052) |
|  | cash-for-care benefit (ref. in-kind benefits) | -0.003  (0.016) | 0.016  (0.017) | 0.003  (0.011) | 0.021^***^  (0.006) | 0.053  (0.035) | 0.029  (0.040) | 0.209^***^  (0.054) |
|  | LTC beds (p. 1,000) | 0.002^**^  (0.001) | -0.000  (0.001) | 0.002^***^  (0.001) | -0.000  (0.000) | 0.002  (0.002) | 0.013^***^  (0.002) | 0.000  (0.003) |
|  |  |  |  |  |  |  |  |  |
|  | Pension generosity |  |  |  |  |  |  |  |
|  | net replacement rate | 0.000  (0.000) | 0.001  (0.000) | 0.001^**^  (0.000) | 0.000  (0.000) | 0.003^***^  (0.001) | 0.004^***^  (0.001) | 0.003^**^  (0.001) |
|  |  |  |  |  |  |  |  |  |
|  | Macroeconomic factors |  |  |  |  |  |  |  |
|  | consumer price index(personal care) | 0.001  (0.002) | 0.005^**^  (0.002) | -0.004^***^  (0.001) | -0.001  (0.001) | 0.012^***^  (0.003) | -0.018^***^  (0.005) | -0.006  (0.006) |
|  | GDP p. capita (in PPS) | -0.122^***^  (0.041) | -0.190^***^  (0.050) | 0.014  (0.026) | 0.009  (0.020) | -0.650^***^  (0.124) | -0.067  (0.110) | -0.058  (0.116) |
|  | female labor market part. (%) | -0.002 (0.002) | -0.002  (0.002) | -0.003^***^  (0.001) | 0.001  (0.001) | -0.009^***^  (0.004) | -0.016^***^  (0.003) | 0.005  (0.005) |
|  | N | 35,547 | 6,959 | | | 35,547 | | |

Note: (Bootstrapped) standard errors are in parentheses ^*^ *p* < 0.10, ^**^ *p* < 0.05, ^***^ *p* < 0.01

Table S.16: Probability of care need (1+ADL) and utilization – individual- and country-level average marginal effects (excluding wealth)

|  |  | (Part I)  **Need for personal care** | | (Part II)  **Care use conditional on need** | | **Unconditional**  **care use** | |  |
| --- | --- | --- | --- | --- | --- | --- | --- | --- |
|  |  | *(1)*  *1+ADL* | | *(2)*  *care utilization* | | *(3)*  *care utilization* | | |
| **Individual-level characteristics** |  |  |  |  |  |  |  | |
|  | Socioeconomic status |  |  |  |  |  |  | |
|  |  |  |  |  |  |  |  | |
|  | *Human resources*  *(ref. primary education)* |  |  |  |  |  |  | |
|  | secondary education | -0.038^***^ | (0.005) | -0.049^*^ | (0.019) | -0.179^***^ | (0.027) | |
|  | tertiary education | -0.073^***^ | (0.006) | -0.087^***^ | (0.025) | -0.372^***^ | (0.037) | |
|  |  |  |  |  |  |  |  | |
|  | *Social resources* |  |  |  |  |  |  | |
|  | has spouse/partner | -0.017^***^ | (0.004) | 0.178^***^ | (0.016) | 0.084^***^ | (0.019) | |
|  | has children | -0.018^***^ | (0.006) | -0.026 | (0.025) | -0.094^***^ | (0.035) | |
|  |  |  |  |  |  |  |  | |
|  | Predisposing factors |  |  |  |  |  |  | |
|  | age | 0.002^***^ | (0.001) | 0.002 | (0.010) | 0.012^***^ | (0.003) | |
|  | age^2^ | 0.00013^***^ | (0.000) | 0.00027^***^ | (0.000) | 0.00078^***^ | (0.000) | |
|  | gender (female) | -0.008^**^ | (0.003) | -0.017 | (0.015) | -0.045^**^ | (0.020) | |
|  |  |  |  |  |  |  |  | |
|  |  |  |  |  |  |  |  | |
| **Country-level characteristics** | LTC policy |  |  |  |  |  |  | |
|  | means testing (ref. none) | -0.008 | (0.015) | -0.062 | (0.038) | -0.088^***^ | (0.026) | |
|  | cash-for-care benefits  (ref. in-kind benefits) | 0.025^*^ | (0.013) | 0.014 | (0.036) | 0.117^***^ | (0.023) | |
|  | LTC beds (p. 1,000) | 0.001 | (0.001) | 0.003 | (0.002) | 0.005^***^ | (0.001) | |
|  |  |  |  |  |  |  |  | |
|  | Pension generosity |  |  |  |  |  |  | |
|  | net replacement rate | -0.000 | (0.000) | 0.003^***^ | (0.001) | 0.001 | (0.001) | |
|  |  |  |  |  |  |  |  | |
|  | Macroeconomic factors |  |  |  |  |  |  | |
|  | consumer price index (personal care) | 0.003^*^ | (0.002) | -0.004 | (0.004) | 0.008^***^ | (0.002) | |
|  | GDP p. capita (in PPS) | -0.054 | (0.035) | -0.166^*^ | (0.093) | -0.362^***^ | (0.067) | |
|  | female labor market part. (%) | -0.001 | (0.001) | -0.005 | (0.004) | -0.009^***^ | (0.002) | |
|  | N | 35,547 | | 4,246 | | 35,547 | | |

Note: (Bootstrapped) standard errors are in parentheses ^*^ *p* < 0.10, ^**^ *p* < 0.05, ^***^ *p* < 0.01

1. W*e provide results using the Global Activity Limitation Indicator (GALI) as indicator for care need. To further control for severity of need we made use of other definitions including, at least two IADL’ or, at least two ADL’. Results of these sensitivity tests can be provided upon request.* [↑](#footnote-ref-1)
